# Impact of sub-basalt thrust systems on the Faroe continental shelf for the late Paleoproterozoic–Cenozoic tectonic evolution of the margin.

**DOI:** 10.12688/openreseurope.18284.2

**Published:** 2024-12-07

**Authors:** Jean-Baptiste P. Koehl, David W. Jolley, Alexander L. Peace, Jhon M. Muñoz-Barrera, Gillian R. Foulger

**Affiliations:** 1 Earth and Planetary Sciences, McGill University Faculty of Science, Montreal, Québec, H3A 0E8, Canada; 2 Geosciences, University of Oslo, Oslo, Oslo, 0371, Norway; 3 Geology and Geophysics, University of Aberdeen School of Geosciences, Aberdeen, Scotland, AB24 3UE, UK; 4 School of Earth, Environment and Society, McMaster University School of Earth Environment & Society, Hamilton, Ontario, L8S 4L8, Canada; 5 National Agency of Hydrocarbon, Bogota, Cundinamarca, 111321, Colombia; 6 Earth Science, University of Bergen, Bergen, Hordaland, 5007, Norway; 7 Earth Sciences, Durham University Department of Earth Sciences, Durham, England, DH1 3LE, UK

**Keywords:** Plate tectonics, fault, shear zone, thrust, orogen, Laxfordian Orogen, Ammassalik Belt, Nagssugtoqidian Orogen, late Paleoproterozoic, Neoproterozoic, Caledonian Orogen, inheritance, Orogenic Bridge Theory, Greenland-Iceland-Faroe Ridge, Seaward-Dipping Reflectors

## Abstract

**Background:**

The Faroe margin in the northeastern Atlantic is segmented by margin-orthogonal, WNW–ESE-striking lineaments extending several hundred kilometers out to the continent–ocean transition. Despite several earlier studies speculating that these features are the product of reactivation of pre-Cenozoic basement-seated structures at depth, the thick Cenozoic volcano-sedimentary sequences deposited along the margin mask the underburden, thus rendering the identification and interpretation of such structures and resolving the pre-Cenozoic history of the area challenging. The present study documents for the first time the existence of margin-orthogonal basement-seated thrust systems and describes their detailed geometry, kinematics, and tectonic evolution.

**Methods:**

We interpreted basement-seated tectonic structures on seismic reflection data from TGS on the Faroe Platform and the Wyville–Thomson and Munkagrunnur ridges using the newly established seismic facies of major thrust systems.

**Results:**

The data show that the Wyville–Thomson Ridge, Munkagrunnur Ridge, and Faroe Platform are cored by WNW–ESE-striking thrust systems hundreds of kilometers long and 30–50 km wide, showing dominantly top-SSW kinematics. The thrusts were reworked into NE–SW-striking folds during the Caledonian Orogeny and controlled the formation of Caledonian thrusts, which in turn controlled the formation of post-Caledonian faults. The pre-Caledonian nature of the WNW–ESE-striking thrusts and their geometry and kinematics suggest a relationship with late Paleoproterozoic Laxfordian shear zones onshore northern Scotland and the continuation of the coeval Nagssugtoqidian Orogen in southeastern Greenland, the Ammassalik Belt. The thrust systems also align with the Tornquist Zone in eastern Europe and the North Sea, thus suggesting either that they controlled the formation of the Tornquist Zone or a possibly much longer (Paleoproterozoic?) tectonic history for the Tornquist Zone.

**Conclusions:**

The Faroe Island margin is crosscut by late Paleoproterozoic Laxfordian–Nagssugtoqidian thrust systems, which controlled further tectonic development of the margin.

## Plain language summary

The Faroe Islands and nearby areas were covered by thick lava flows in the Cenozoic (< 65 million years ago) during the opening of the North Atlantic Ocean. Thus far, the strong seismic signal of the lavas made the study of deeper and older rock units and tectonic structures (> 65 million years old) challenging. In the present work, we describe newly identified, hundred of km long and tens of km wide systems of old cracks in the Earth’s crust in the subsurface around the Faroe Islands. The cracks trend parallel to the Greenland–Iceland–Faroe Ridge and are probably part of an 1.8 billion year old system, which continues onshore northern Scotland and has counterparts in southeastern Greenland formed during the collision of two or more tectonic plates. The newly identified structures controlled the formation of contractional structures formed during the collision of Greenland with Europe ca. 425 million years ago and of subsequent extensional structures. In addition, the present work suggests that the Greenland–Iceland–Faroe Ridge partly consists of ≥ 1.8 million years old continental crust instead of < 65 million years old oceanic crust as previously suggested. This has implications for our understanding of plate tectonics and Earth’s internal dynamics.

The present study shows a new method to investigate the old tectonic history of entire regions using seismic reflection data, in the hope that it will be used more widely by the scientific community in the future to identify and study similar structures worldwide, for example when evaluating earthquake risk and exploring for natural resources such as white and orange hydrogen.

## Introduction

**Figure 1.**
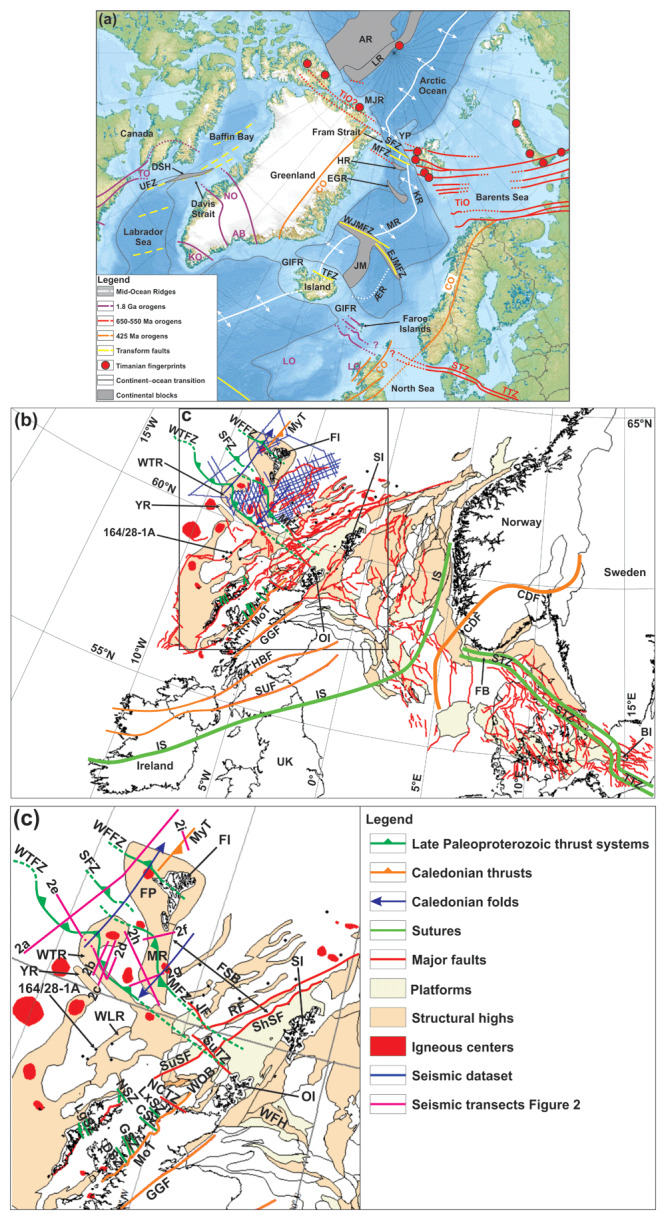
( **a**) Regional map of the Northeast Atlantic Ocean and main structural elements. Notice the alignment of the Tornquist Zone with the major (late Paleoproterozoic?) thrust systems discussed in the present study. Basemap is the International Bathymetric Chart of the Arctic Ocean ( Jakobsson *et al.*, 2012). Abbreviations: AB: Ammassalik Belt; AR: Alpha–Mendeleev Ridge; ÆR: Ægir Ridge; CO: Caledonian Orogen; DSH: Davis Strait High; EGR: East Greenland Ridge; EJMFZ: East Jan Mayen Fault Zone; GIFR: Greenland–Iceland–Faroe Ridge; HR: Hovgård Ridge; JM: Jan Mayen Microcontinent Complex; KO: Ketilidian Orogen; KR: Knipovich Ridge; LO: Laxfordian Orogen; LR: Lomonosov Ridge; MFZ: Molloy Fault Zone; MJR: Morris Jesup Rise; MR: Mohns Ridge; NO: Nagssugtoqidian Orogen; SFZ: Spitsbergen Fault Zone; STZ: Sorgenfrei–Tornquist Zone; TFZ: Tjörnes Fault Zone; TiO: Timanian Orogen; TO: Torngat Orogen; TTZ: Teisseyre–Tornquist Zone; WJMFZ: West Jan Mayen Fault Zone; YP: Yermak Plateau. ( **b**) Structural map of the continental shelf in Scandinavia, the North Sea, the UK, and the Faroe Islands showing the outline of major structures, basins, and highs in the region. Abbreviations: CDF: Caledonian Deformation Front; FB: Farsund Basin; FI: Faroe Islands; GGF: Great Glen Fault; HBF: Highland Boundary Fault; IS: Iapetus Suture; MFZ: Munkagrunnur fault zone; MoT: Moine Thrust; MyT: Mykines thrust; OI: Orkney Islands; SI: Shetland Islands; STZ: Sorgenfrei–Tornquist Zone; SUF: Southern Upland Fault; TTZ: Teisseyre–Tornquist Zone; UK: United Kingdom; WFFZ: West Faroe fault zone; WTFZ: Wyville–Thomson fault zone; WTR: Wyville–Thomson Ridge; YR: Ymir Ridge. ( **c**) Zoom in the study area off the Faroe Islands. The location of ( **c**) is shown as a black frame in ( **b**). Major sutures, fault zones onshore the UK, and deformation fronts are from Pharaoh (1999). The geometry and location of the Sorgenfrei–Tornquist Zone and Teisseyre–Tornquist Zone are from Mazur *et al.* (2015), Phillips *et al.* (2018), and Ponikowska *et al.* (2024). Basins and highs in the northern UK, Shetland Island, and Faroe Island regions are from Johnson *et al.* (2005), Wilson *et al.* (2010), Bird *et al.* (2016), and Jolley *et al.* (2021), from the Norwegian Offshore Directorate and De Luca *et al.* (2023) for the North Sea, and from Vejbæk and Andersen (2002) and Gregersen *et al.* (2022) for the southeastern North Sea and southern Baltic Sea. Paleoproterozoic shear zones onshore northern Scotland are from Coward and Park (1987) and Bergh *et al.* (2012). Abbreviations: CSZ: Canisp Shear Zone; DSZ: Diabaig Shear Zone; FI: Faroe Islands; FP: Faroe Platform; FSB: Faroe–Shetland Basin; GGF: Great Glen Fault; GSZ: Gairloch Shear Zone; JF: Judd Fault; LgSZ: Langavat Shear Zone; LxSZ: Laxford Shear Zone; MFZ: Munkagrunnur fault zone; MR: Munkagrunnur Ridge; NCTZ: North Coast Transfer Zone; NSZ: Ness Shear Zone; MoT: Moine Thrust; MyT: Mykines thrust; OI: Orkney Islands; RF: Rona Fault; SFZ: Suðurøy fault zone; ShSF: Sheltand–Spine Fault; SI: Shetland Islands; SuTZ: Sula Transfer Zone; SuSF: Sula–Sgier Fault; WFFZ: West Faroe fault zone; WFH: West Fladen High; WLR: West Lewis Ridge; WOB: West Orkney Basin; WTFZ: Wyville–Thomson fault zone; WTR: Wyville–Thomson Ridge; YR: Ymir Ridge.

The Faroe Islands lie along the northwestern part of the European continental shelf ( Figure 1a), which was rifted away from its conjugate counterpart in southeastern Greenland in the Cenozoic, after repeated extensional events had affected the margin. These include Devonian collapse, Permian–Triassic, Cretaceous, and early Cenozoic events ( Bird *et al.*, 2016; Coward *et al.*, 1989; Jolley *et al.*, 2021; Wilson *et al.*, 2010). A major outstanding issue with the pre-Cenozoic geology of the area is the difficulty in imaging rock units below the thick lava flows ( Ólavsdóttir *et al.*, 2016) and the lack of studies showing the seismic character of metamorphosed basement rocks. For this study, we benefitted from reprocessed 2D seismic reflection data from TGS (survey OF95RE11) and from several recent studies focusing on local- (a few hundreds of meters high and wide) to regional-scale structures (up to hundreds of kilometers long, tens of kilometers thick) within metamorphosed basement rocks ( Koehl, 2020; Koehl, 2021; Koehl, 2024a; Koehl *et al.*, 2022; Koehl *et al.*, 2023a; Koehl & Stokmo, 2024).

Previously, late Paleocene–Miocene compression related to anomalous ridge-push along the extinct Ægir Ridge ( Boldreel & Andersen, 1993; Boldreel & Andersen, 1998) and/or (Precambrian?) preexisting structures and transfer zones ( Johnson *et al.*, 2005; Kimbell *et al.*, 2005), or early Paleocene rifting ( Ziska & Varming, 2008) were proposed as causes for the formation of margin-oblique to margin-orthogonal structural elements such as the Wyville–Thomson, Ymir, and Munkagrunnur ridges and the Faroe Platform. However, the nature of the rocks (metamorphosed basement rocks or inverted sedimentary basin) at the core of these structural highs was thus far uncertain and the transition between these structures poorly understood ( Ólavsdóttir *et al.*, 2016). The present study reveals the nature of the rocks within these highs and proposes a much older origin and tectonic history, explaining both previously inferred Cenozoic contractional reactivation and inherited basement-seated transfer zones at these margin-oblique/orthogonal structural elements. Our findings also invalidate an early Paleocene rift-related origin for these structures.

The present study extends the interpretation of old (Paleoproterozoic) orogenic belts and continental crust farther offshore. Hence, it has direct implications for the orogenic bridge theory proposed by Koehl and Foulger (2024) and for plate tectonics in general. For example, it suggests that tectonic plates are less mobile over time than previously suggested. It also has implications for regional correlations of old (Paleoproterozoic) orogens and fold-and-thrust belts. Major implications also include the use of seismic reflection imaging to map contractional ductile shear zones and thrust systems, which is now proven at various margins and will, hopefully, be widely used in the coming years.

## Geological setting

### Wyville–Thomson and Ymir ridges

The Wyville–Thomson and Ymir ridges are elongate, respectively 30–50 km and < 10 km wide, WNW–ESE-striking high southwest of the Faroe Islands ( Figure 1b). The ridges are capped by Paleogene lava flows and Cenozoic sedimentary deposits, which thin over the ridges, and are onlapped by (Cretaceous?) sedimentary rocks underlying the Paleocene lavas (e.g., Johnson *et al.*, 2005, their figure 6). Notably, previous studies showed that Eocene sedimentary units are folded, whereas Oligocene sedimentary successions onlap the ridges. These lines of evidence were used to suggest that both ridges were topographic highs during most of the Cenozoic. Previously proposed formation mechanisms include the reactivation of WNW–ESE-striking transfer faults and/or various episodes of post-breakup contraction, including in the late Eocene–mid Oligocene ( Boldreel & Andersen, 1993; Boldreel & Andersen, 1998; Johnson *et al.*, 2005; Kimbell *et al.*, 2005; Kimbell *et al.*, 2016; Smallwood, 2008; Waddams & Cordingley, 1999). Boldreel and Andersen (1993) proposed that the ridges initiated from the inversion of an extensional fault, whereas Kimbell *et al.* (2005) suggested that the ridges are part of a Cenozoic ramp-anticline complex.

### Munkagrunnur Ridge

The Munkagrunnur Ridge is a N–S-trending topographic high south of the Faroe Islands, which bends into a NW–SE trend towards the Wyville–Thomson Ridge in the south and bounds the Faroe–Shetland Basin in the east ( Figure 1c). Formation involving several stages of Cenozoic (e.g., late Eocene–mid Oligocene) post-breakup contraction was proposed previously, i.e., similar to the Wyville–Thomson and Ymir ridges ( Johnson *et al.*, 2005; Smallwood, 2008; Stoker *et al.*, 2015). Detailed sequence stratigraphic investigations even suggested repeated (up to eight discrete, < 1 Myr) episodes of tectonic uplift of the ridge during the mid-Eocene ( Ólavsdóttir *et al.*, 2010). An origin of the ridge as a drape fold between two synclines has also been considered ( Stoker *et al.*, 2015).

### Faroe platform

The Faroe Platform is a structural high located at the northwestern edge of the European continental shelf encompassing the Faroe Islands. It consists of 20–46 km of continental crust ( Funck *et al.*, 2017; Richardson *et al.*, 1998; Figure 1c). Despite its shallow character, the 3–9 km thick cover of Paleogene lava flows and sedimentary rocks, which crop out on the seabed and on the Faroe Islands, have thus far made it difficult to resolve the pre-rift evolution of the platform ( Ólavsdóttir *et al.*, 2016; Ólavsdóttir *et al.*, 2021; Richardson *et al.*, 1998). Nevertheless, forward gravity modelling suggests that the lava flows are underlain by two sedimentary basins ( Ólavsdóttir *et al.*, 2021).

## Methods

**Figure 2.**
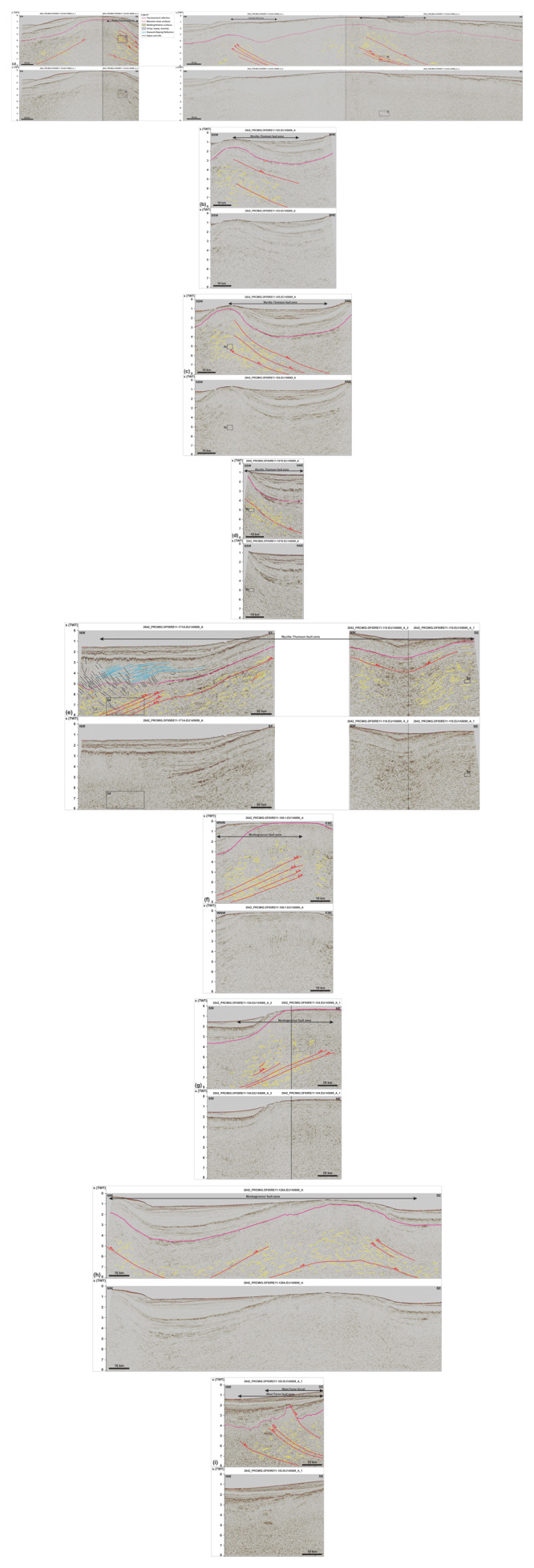
Seismic reflection data off the Faroe Island continental shelf. See Figure 1c for location. Data courtesy of TGS. ( **a**) NNE–SSW-trending seismic transect west of the Faroe Islands showing the occurrence of major NNE-dipping, top-SSW thrust systems below Cenozoic lavas off the Faroe Islands. The thrust systems consist of major mylonitic shear surfaces (red lines) and of tightly folded bedding or foliation surfaces (yellow lines). Relatively small (kilometer to hundreds of meters wide) features of interest include asymmetric (up to isoclinal recumbent) folds, duplexes, and antiformal thrust stacks. The vergence of asymmetric fold structures (and mylonitic shear surfaces) is opposite on either side of major ridges and highs, e.g., at Wyville–Thomson Ridge, suggesting limited amounts of movement. There are Z-shaped reflections in the lower part of the Suðurøy and West Faroe fault zones suggesting extensional reactivation of the fault zones. ( **b**), ( **c**) and ( **d**) NE–SW-trending seismic transects showing the continuation of the top-SSW Wyville–Thomson fault zone at Wyville–Thomson Ridge. ( **e**) WNW–ESE-trending seismic section along the Wyville–Thomson Ridge showing tens of kilometers wide, open, NNE–SSW-striking macrofolds deforming the top-SSW Wyville–Thomson fault zone. The opposite sense of shear is indicated by asymmetric folds and minor brittle thrusts on either limbs of the macrofolds, which suggests limited amount of tectonic displacement. In the northwest, the section displays gently northwest-dipping, moderate-amplitude reflections (blue lines), curving-downward reflections (black lines), and southeast-dipping disruption surfaces (black lines) interpreted respectively as SDRs, saucer-shaped sills, and dykes and sills. The later crosscut the folded Wyville–Thomson fault zone. The Wyville–Thomson fault zone and related asymmetric folds extend below and northwest of the SDRs suggesting that the Iceland–Faroe Ridge consists (at least partly) of continental crust. ( **f**) ENE–WSW-trending seismic section at the Munkagrunnur Ridge showing asymmetric folds indicating top-east kinematics along the Munkagrunnur fault zone. The Z-shaped reflections suggest extensional reworking of the fault zone. ( **g**) NE–SW-trending seismic transect at Munkagrunnur Ridge showing the dominance of top-NNE kinematic indicators (e.g., NNE-verging folds and top-NNE minor brittle thrusts) along the Munkagrunnur fault zone. ( **h**) NW–SE-trending transect along the Munkagrunnur Ridge showing the reworking of the Munkagrunnur fault zone by a tens of kilometers wide, NNE–SSW-striking, SSW-plunging macrofold with opposite sense of shear on either flanks. ( **i**) Folded portion of the West Faroe fault zone that was overprinted by a top-northwest Caledonian thrust. The listric, post-Caledonian, brittle, normal fault, which offsets the Top-basement reflection by ca. 1 second (TWT) merges with the top-northwest thrust at depth suggesting it formed along preexisting zones of weakness in the crust.

We used seismic reflection data from TGS to interpret basement-seated contractional ductile shear zones and thrust systems around the Faroe Islands (survey OF95RE11). Our interpretation is based on the previous detailed description of the internal geometry of thrust systems by Koehl *et al.* ( 2022; 2023a), Koehl (2024a), and Koehl and Stokmo (2024). Mylonitic thrusts were interpreted using previous works by Christensen (1965), Fountain *et al.* (1984), and Hurich *et al.* (1985). In addition, we used modern examples of mylonitic thrusts in metamorphosed basement rocks on seismic data to refine our interpretation (e.g., Fazlikhani *et al.*, 2017; Hedin *et al.*, 2016; Koehl *et al.*, 2018; Phillips *et al.*, 2016). High-resolution versions of the data ( Figure 2a–i) are available on DataverseNO ( https://doi.org/10.18710/780M9P).

The data frequency (c. 40 Hz; Nicholson, 2012 her figure 1) and seismic velocity within Paleoproterozoic metamorphosed basement rocks (overall 6200–6400 m.s ^-1^, i.e., c. 6300 m.s ^-1^; Bamford, 1979; Luckett & Baptie, 2015; Watson & Dunning, 1979 their figures 4, 10, and 11) and in mylonites (up to 6700 m.s ^-1^; e.g., Kästner *et al.*, 2020 their table 2) indicate that the vertical resolution of the seismic data (1/4 of the wavelength) is c. 39 m (6300/40/4). In places, the vertical resolution of seismic data may be as good as 1/32 of the wavelength ( Kallweit & Wood, 1982; Li & Zhu, 2000), i.e., up to c. 5 m in the present case (6300/40/32). The horizontal resolution of the data at depth is a function of depth and the wavelength ( Geldart & Sheriff, 2004) and is c. 627 m at a 5000 m depth ((5000 × (6300/40/2)) ^1/2^). Since the studied asymmetric folds within major shear zones and thrust systems are generally > 500 meters wide and > 150 meters thick, they are well within the vertical and horizontal resolution of the seismic dataset even at high depth (> 5000 m). Noteworthy, the horizontal resolution of the data at a depth of 2900 m (termination of well 164/28-1A) is c. 478 m ((2900 × (6300/40/2)) ^1/2^). See Supplement 5 in Koehl (2024b) attached to the interpretation in Koehl and Stokmo (2024) for more information on the resolvability of the targeted intra-shear-zone structures such as hundreds-of-meter- to kilometer-scale asymmetric folds on seismic reflection data.

Structures in overlying post-Caledonian sedimentary and igneous rocks only mildly (if at all) rework the studied pre-Cretaceous structures and, thus, have little impact on the geometry of the studied structures. Post-Caledonian structures were therefore not investigated, except where they showed a clear relationship with the studied basement-seated structures (e.g., merging, truncating, and/or reworking).

Our interpretation was tied to exploration wells 164/28-1A west of the West Lewis Ridge ( Figure 1c), which terminated at a depth of c. 2900 m in Cretaceous sedimentary successions (see Jolley *et al.*, 2021, their figure 19 for the tie). The well also penetrated a c. 400 meters thick volcanoclastic succession including hyaloclastite and lava flows ( Jolley *et al.*, 2021). The main Top-basement unconformity was interpreted as a major unconformity with onlap of Cretaceous sedimentary rocks onto the Wyville–Thomson Ridge throughout the study area.

We used Petrel 2021.3 to interpret seismic reflection data, and CorelDraw 2017 to design the figures. Alternative open-source software are OpendTect and GIMP respectively.

## Results

### Observations

***Wyville–Thomson Ridge.*** Basement rocks at the WNW–ESE-striking Wyville–Thomson Ridge present several types of structures below the Top-basement unconformity, all of which are characteristic of major contractional shear zones and thrust systems. These include mylonitic shear surfaces (e.g., Fountain *et al.*, 1984; Hurich *et al.*, 1985; Phillips *et al.*, 2016; Reeve *et al.*, 2013), asymmetric folds (e.g., Koehl, 2024a; Koehl *et al.*, 2022; Koehl *et al.*, 2023a; Koehl *et al.*, 2023b; Koehl & Stokmo, 2024), duplexes (e.g., Koehl, 2021; Koehl, 2024a; Koehl *et al.*, 2022; Koehl *et al.*, 2023b; Koehl & Stokmo, 2024), and minor brittle thrusts (e.g., Brewer *et al.*, 1980; Brewer *et al.*, 1981; Koehl, 2021; Koehl *et al.*, 2022; Koehl *et al.*, 2023b; Koehl & Stokmo, 2024; O’Connor, 1992; Shaw *et al.*, 1999). These are further described below.

**Figure 3.**
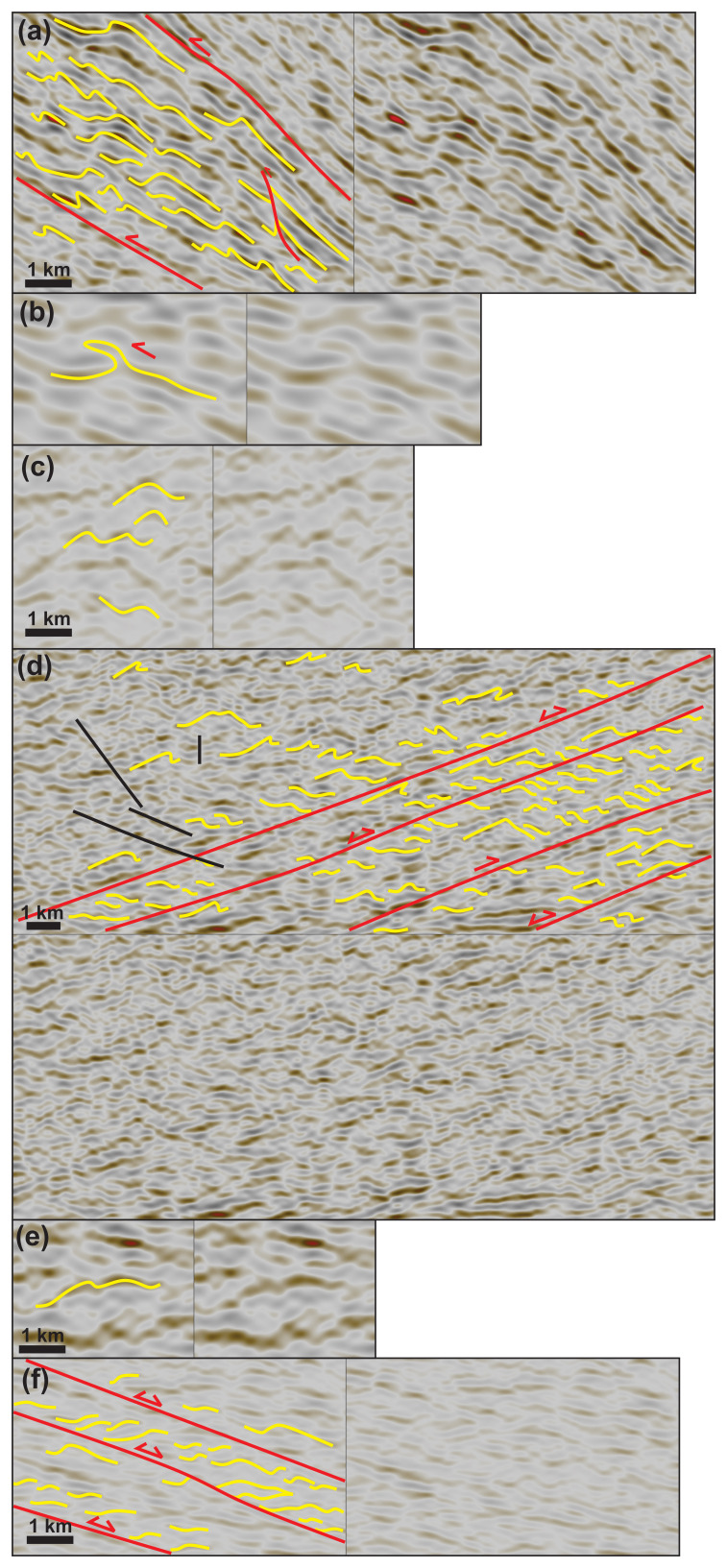
Zoom in specific structures on seismic reflection data. See Figure 2 for legend. All figure insets show interpreted data on the left-hand side and uninterpreted data on the right-hand side, except for ( **d**), which shows interpreted data up and uninterpreted data down. Data courtesy of TGS. ( **a**) Forward-dipping duplexes (see Koehl, 2021 for definition) consisting of SSW-verging asymmetric folds, minor top-SSW brittle thrusts, and antiformal stacks (yellow lines) bounded upwards and downwards by major mylonitic shear surfaces (red lines) along the Wyville–Thomson fault zone. All the structures consistently indicate top-SSW kinematics. See location in Figure 2a. ( **b**) Isoclinal recumbent fold indicating top-SSW kinematics along the Wyville–Thomson fault zone. See location in Figure 2d. ( **c**) Symmetric folds within the core of the Wyville–Thomson Ridge suggesting limited movement. See location in Figure 2c. ( **d**) Southwest-verging asymmetric folds and down-northwest extensional duplexes suggesting extensional reactivation of folded WNW–ESE-striking (late Paleoproterozoic) thrust systems. The folded and reactivated/overprinted thrust systems are crosscut by southeast-dipping (Cenozoic) sills and dykes (black lines). See location in Figure 2d. ( **e**) Symmetric folds within the core of NNE–SSW-striking (Caledonian) macrofolds along strike the Wyville–Thomson fault zone. See location in Figure 2e. ( **f**) Z-shaped reflections within WNW–ESE-striking thrust systems. The SSW-verging asymmetric folds (up) are juxtaposed with Z-shaped extensional duplexes across (down) a major NNE-dipping mylonitic shear surface (red line) suggesting down-NNE extensional reactivation of top-SSW thrust systems. See location in Figure 2a.

In NE–SW-trending cross section, basement rocks at the Wyville–Thomson Ridge consist of numerous undulating, upward-convex, low- to moderate-amplitude seismic reflections with an undulation wavelength of a few hundred meters to a few kilometers ( Figure 2a–i). Most of the undulating reflections are asymmetric and typically show a long, gently-dipping edge and a short, steeply-dipping edge ( Figure 2a–d and Figure 3a). In places, these asymmetric reflections display a tight hinge zone with parallel edges ( Figure 3b). A few undulating upward-convex reflections are symmetric and are found at the center of the ridge ( Figure 3c).

On the northeastern flank of the Wyville–Thomson Ridge, numerous asymmetric reflections lean towards the south-southwest, i.e., showing a long, gently-NNE-dipping edge and a short, steeply-SSW-dipping edge ( Figure 2a–d and Figure 3a–b). Subsidiary undulating reflections on the southwestern flank of the ridge show opposite characteristics with a long, gently-SSW-dipping southwestern edge and a short, steeply-NNE-dipping northeastern edge ( Figure 2a–c).

**Figure 4.**
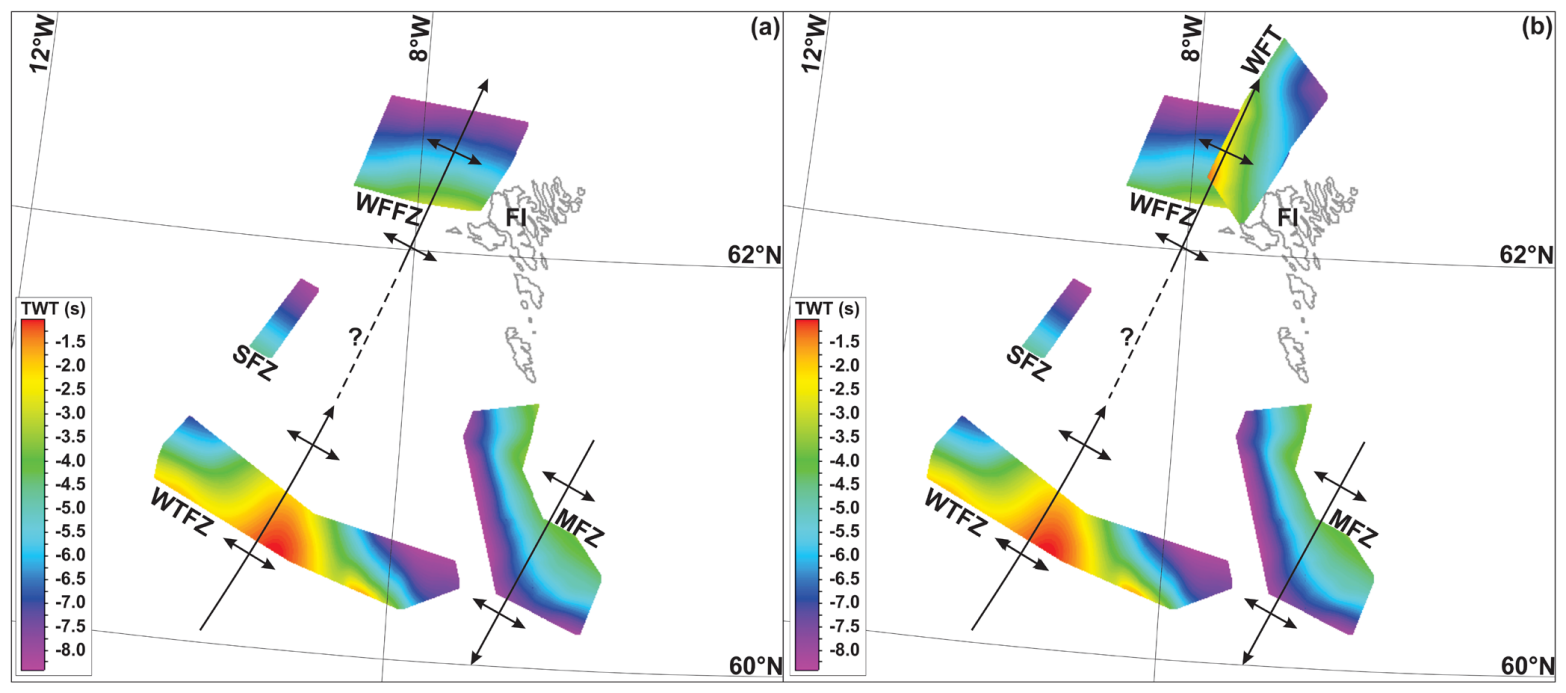
Time (TWT) maps of the WNW–ESE-striking thrust systems off the Faroe Islands without ( **a**) and with ( **b**) Caledonian thrusts (West Faroe thrust). There is apparent folding of the thrust systems into tens of kilometers wide, NNE–SSW-striking, dominantly NNE- and subsidiarily SSW-plunging macrofolds and the formation of Caledonian thrusts along the limbs of the Caledonian macrofolds. Abbreviations: FI: Faroe Islands; MFZ: Munkagrunnur fault zone; SFZ: Suðurøy fault zone; WFFZ: West Faroe fault zone; WFT: West Faroe thrust; WTFZ: Wyville–Thomson fault zone.

In places, some asymmetric undulating reflections are arranged in packages separated by planar, 2–8 seconds (TWT) deep, gently-NNE-dipping, moderate- (in places high-) amplitude disruption surfaces ( Figure 2a–i). These disruption surfaces are traced for tens of kilometers in cross section and for at least 150 km along the strike of the Wyville–Thomson Ridge ( Figure 1c and Figure 4a). In addition, minor, a few hundred meters to a few kilometers, high-angle disruption surfaces mildly offset asymmetric reflections in a reverse fashion ( Figure 3a).

In NW–SE-trending along-strike section, the major, hundreds of kilometers long disruption surfaces are folded into up to 100 km wide, NE–SW-striking open folds ( Figure 2e). These NE–SW-striking folds are confirmed by the depth map (i.e., map-view geometry) of the main disruption surface, which also shows that these major open folds plunge moderately to the north-northeast, displaying dome- and trough-shaped geometries ( Figure 4a).

In the northwest, NW–SE-trending sections show a series of thick, moderate-amplitude, gently-northwest-dipping reflections between 3 and 5 seconds (TWT; see blue lines in Figure 2e), which have been previously interpreted as Seaward-Dipping Reflectors (SDRs; Davison *et al.*, 2010; Parson *et al.*, 1988; Smythe, 1983; Spence *et al.*, 1989). Farther northwest, these are truncated by numerous, moderately- to steeply-southeast-dipping disruption surfaces within low- to moderate-amplitude rock units (see planar black lines in Figure 2e). These disruption surfaces are observed up to a depth of 7.5 seconds (TWT) and terminate below a layer of high-amplitude, flat-lying sedimentary strata crosscut by multiple high-amplitude, U-shaped reflections at a depth of 2.5–3 seconds (TWT) interpreted as saucer-shaped sills (see U-shaped black lines in Figure 2e).

At a depth ≥ 6 seconds (TWT) below the moderately- to steeply-southeast-dipping disruption surfaces and gently-northwest-dipping SDRs, upward-convex reflections similar to those within the Wyville–Thomson Ridge are arranged in packages of dominantly southeast-leaning and locally Z-shaped reflections separated by gently-northwest-dipping disruption surfaces ( Figure 3d). Locally, the upward-convex reflections display intermediate geometries between Z-shaped and southeast-leaning ( Figure 3d).

***Munkagrunnur Ridge.*** South of the Faroe Islands along the western flank of the Munkagrunnur Ridge ( Figure 1c), the data shows that basement rocks are dominated by a few hundred meters to a few kilometers wide, asymmetric reflections leaning to the east ( Figure 2f). Farther south, the reflections lean to the north-northeast ( Figure 2g). Similarly, a major, 4–8 seconds (TWT) deep disruption surface is observed in the area and displays a comparable, c. 70° map-view change in orientation, i.e., bending from a west-dipping geometry in the north, just south of the Faroe Islands, to a SSW-dipping geometry at the southern tip of the Munkagrunnur Ridge ( Figure 1c).

In NW–SE-striking sections, the major disruption surface appears folded into a major, ≥ 70 km wide, southwest-plunging antiform, and undulating reflections are dominantly symmetrical within the Munkagrunnur Ridge ( Figure 2h), i.e., similar to the disruption surfaces and undulating reflection within the Wyville–Thomson Ridge ( Figure 2e and Figure 3e).

***Faroe Platform.*** West and southwest of the Faroe Islands, basement rocks show asymmetric reflections in NE–SW-trending cross sections comparable to those at Wyville–Thomson Ridge ( Figure 2a). Additional features of interest are packages of Z-shaped reflections separated by planar, NNE-dipping disruption surfaces ( Figure 3d and f) and 30–40 km wide macrofolds of the Top-basement reflection similar to that observed at the Wyville–Thomson Ridge ( Figure 2a). In map view, the main disruption surfaces appears mildly folded into a ≥ 50 km wide, NNE-plunging antiform similar to that observed at the Wyville–Thomson Ridge ( Figure 4a).

On the southeastern limb of this major NNE-plunging fold, asymmetric, undulating, low- to moderate-amplitude reflections lean to the northwest and disruption surfaces dip gently to moderately to the southeast ( Figure 2i), i.e., similarly to asymmetric undulating reflections and disruption surfaces in the southeastern limb of the major NNE-plunging antiform at Wyville–Thomson Ridge ( Figure 2e). In addition, the southeast-dipping disruption surfaces on the Faroe Platform deepen from a c. 2.0–4.0 seconds (TWT) depth in the southwest to a c. 4.25–7.25 seconds (TWT) depth in the northeast, i.e., following the attitude of the NNE-plunging antiform on the Faroe Platform ( Figure 4a–b).

North of the Faroe Islands, a major, high-angle, southeast-dipping, listric disruption surface bounds thickened wedges of post-Caledonian sedimentary rocks against metamorphosed basement rocks consisting of northwest-leaning asymmetric reflections and crosscut by moderately- to gently-southeast-dipping disruption surfaces ( Figure 2i). In the upper part, the high-angle disruption surface offsets discrete reflections by c. 0.1 second (TWT) in a normal fashion ( Figure 2i). At depth, the high-angle disruption surface dips moderately to gently and parallels major southeast-dipping disruption surfaces within basement rocks ( Figure 2i).

### Interpretation

***Magmatic features.*** In the northwest, the moderately southeast-dipping disruption surfaces crosscutting the SDRs and terminating below the saucer-shaped sills do not show any offset of the truncated features ( Figure 2e). A tectonic origin is therefore unlikely. Given their occurrence below a system of saucer-shaped sills and absence farther southeast and their planar and gently- to moderately-dipping geometry ( Figure 2e), they are interpreted as a magmatic feeder system of sills and dykes related to the rifting of the northeastern Atlantic.

***WNW–ESE-striking thrust systems.*** Asymmetric undulating reflections occur as packages consistently displaying a long and a short limb (e.g., long northeastern and short southwestern limb on the northeastern flank of Wyville–Thomson Ridge; Figure 2a) and are in places crosscut and offset by minor, reverse, high-angle disruption surfaces ( Figure 2a and Figure 3a) and by major, moderately dipping disruption surfaces ( Figure 2a–d). These features are typical of major thrust systems, both in the field ( Bell & Hammond, 1984; Fossen & Holst, 1995; Nabavi and Fossen, 2021 their figure 2f; Platt, 1983) and on seismic data (e.g., Koehl *et al.*, 2022; Koehl *et al.*, 2023a; Koehl *et al.*, 2023b; Koehl, 2024a; Koehl & Stokmo, 2024; Figure 2a–i and Figure 3a–f). Thus, the asymmetric reflections are interpreted as folded (bedding? foliation?) surfaces indicating the sense of shear and direction of tectonic transport within metamorphosed basement rocks, and minor, high-angle and major moderately-dipping disruption surfaces as brittle thrusts and contractional mylonitic shear zones within major thrust systems respectively ( Fountain *et al.*, 1984; Hurich *et al.*, 1985; Koehl *et al.*, 2022; Phillips *et al.*, 2016). Major thrust systems in the study area include the NNE-dipping Wyville–Thomson, Suðurøy, and West Faroe fault zones, and the SSW-dipping Munkagrunnur fault zone ( Figure 1b–c and Figure 2a and g).

In places, asymmetric reflections show limbs with the same dip direction and/or parallel to one another, thus suggesting recumbent to isoclinal geometries ( Figure 3b). In addition, in places, asymmetric folds are arranged in packages ( Figure 3a) separated by major WNW–ESE-striking mylonitic shear zones ( Figure 2a–i and Figure 4a). These packages of asymmetric folds are interpreted as contractional, forward-dipping duplexes (see Koehl, 2021 for definition), some of which possibly evolved into antiformal thrust stacks ( Figure 3a; Boyer & Elliott, 1982).

**Figure 5.**
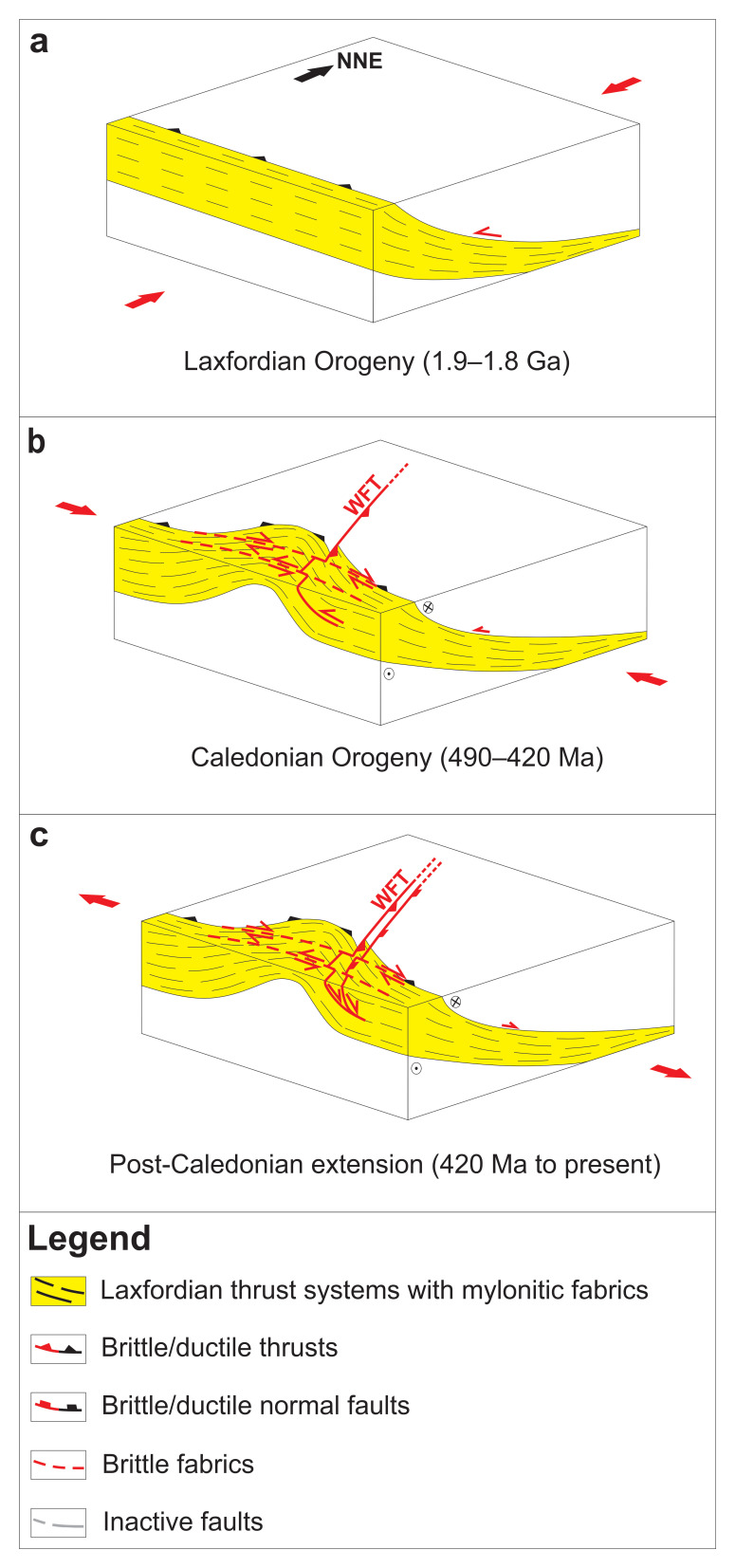
( **a**) Formation of mylonitic, WNW–ESE-striking (dominantly top-SSW) thrust systems during the late Paleoproterozoic Laxfordian (–Nagssugtoqidian) Orogen. ( **b**) Reworking of the thrust systems into tens to hundreds of kilometers wide, NNE–SSW-striking macrofolds during the Caledonian Orogeny and formation of NNE–SSW-striking mylonitic Caledonian thrusts and shear zones (e.g., West Faroe thrust – WFT) on the limbs of folded Paleoproterozoic thrust systems. ( **c**) Post-Caledonian (i.e., Devonian–Permian and possibly Mesozoic–Cenozoic) reactivation and overprinting of Laxfordian thrust systems, including formation of listric brittle normal faults merging with Caledonian and folded Paleoproterozoic thrust systems at depth. Note that the mapped thrust systems were probably reactivated/overprinted during late Neoproterozoic and potentially Cenozoic contractional events.

Overall in the study area, asymmetric folds indicate a dominant top-SSW ( Figure 5a) and subsidiary top-NNE transport directions in NE–SW-striking cross section ( Figure 2a–i and Figure 3a–f). More specifically, at Wyville–Thomson Ridge and on the Faroe Platform ( Figure 1c), asymmetric folds are SSW-verging in the northeastern and NNE-verging in the southwestern flanks of major, open, 30–40 km wide, WNW–ESE-striking macrofolds, which affect the Top-basement reflection and overlying Mesozoic–early Cenozoic volcanosedimentary successions ( Figure 2a–c). The opposite kinematics of the asymmetric folds suggest that they may have formed as parasitic folds. Together with the occurrence of symmetric folds within the hinge of the WNW–ESE-striking macrofolds ( Figure 2a–c and Figure 3c), this suggests an overall limited amount of tectonic movement (probably up to a few tens of km).

***NE–SW-striking Caledonian folds and thrusts.*** In NW–SE-trending along-strike section, the WNW–ESE-striking thrust systems and mylonitic shear zones are folded into open, up to 100 km wide, NE–SW-striking macrofolds that also involve both basement and Mesozoic–early Cenozoic volcanosedimentary successions ( Figure 2e and Figure 5b). This is illustrated for example by the switch in dominant structural strike at the Munkagrunnur Ridge, i.e., top-east folds and shear zones in the west and top-NNE in the south ( Figure 2f–g). These macrofolds include possible southeast- and northwest-verging asymmetric (parasitic?) folds and associated duplexes and minor brittle thrusts ( Figure 2e) and their initiation must postdate the formation of WNW–ESE-striking thrust systems. Similarly to WNW–ESE-oriented structures, asymmetric folds on the limbs of, NE–SW-striking macrofolds also show opposite vergence (top-southeast and top-northwest; Figure 2e), thus also limiting tectonic movements to possibly a few tens of km.

North of the Faroe Islands, the northwest-leaning asymmetric folds and related gently- to moderately-southeast-dipping disruption surfaces suggest the occurrence of a shallow (up to 2.0 seconds TWT) top-northwest shear zone in basement rocks ( Figure 2i and Figure 5b). The shallow character and northeastward deepening geometry of the shear zone, i.e., mimicking the attitude of the underlying, WNW–ESE-striking West Faroe fault zone at depth (i.e., southeast-dipping because folded into a NNE-plunging macrofold) suggests that the West Faroe fault zone controlled the formation of the top-northwest shear zone ( Figure 2i, Figure 4a–b, and Figure 5b).

The top-northwest sense of shear indicated by the northwest-verging folds is comparable to that of the Moine Thrust in northern Scotland ( Coward, 1980; Coward, 1990; Coward *et al.*, 1980; McClay & Coward, 1980; Ramsay, 1969; Soper & Wilkinson, 1975) and in adjacent offshore areas (e.g., Bird *et al.*, 2016). In addition, the seismic geometry of the top-northwest shear zones are similar to that of the Moine Thrust ( Bird *et al.*, 2016). This suggests that the top-northwest shear zone and related asymmetric folds and macrofolds ( Figure 2e and Figure 3e) initiated during the Caledonian Orogeny ( Figure 5b). We name this fault the West Faroe Thrust.

***Post-Caledonian normal faults and reactivation.*** The high-angle disruption north of the Faroe Islands offsets post-Caledonian sedimentary successions in a normal fashion and is therefore interpreted as a normal fault. The fault merges with the top-northwest West Faroe Thrust at depth ( Figure 2i and Figure 5c). A similar relationship was documented along the offshore continuation of the Moine Thrust in the West Orkney Basin, where post-Caledonian (Devonian–Permian) brittle normal faults merge at depth with the Moine Thrust ( Bird *et al.*, 2016). In addition, folded portions of the WNW–ESE-striking thrust systems west of the Faroe Islands (e.g., Wyville–Thomson fault zone below the SDRs) show Z-shaped reflections within duplex structures ( Figure 2e). These indicate that folded portions of WNW–ESE-striking thrust systems were inverted during post-Caledonian extension ( Figure 3d and f), and that Caledonian and pre-Caledonian structures controlled the formation of subsequent post-Caledonian normal faults in the study area ( Figure 5c).

## Discussion

### Timing of formation of WNW–ESE-striking thrust systems

The inversion of WNW–ESE-striking thrust systems and related structures ( Figure 2a and e and Figure 3d and f) and their controlling relationship over Caledonian folds and thrusts and post-Caledonian normal faults ( Figure 2i and Figure 4a–b) imply a pre-Caledonian origin for WNW–ESE-structures. Their strike possibly indicates that they formed during an episode of NE–SW-oriented contraction. Here, we review possible origins for the WNW–ESE-striking thrust systems off the Faroe Islands.

***Late Paleoproterozoic origin.*** The 240 km wide, WNW–ESE-striking Ammassalik Belt in southeastern Greenland is the southeastern continuation of the late Paleoproterozoic Nagssugtoqidian Orogen in western Greenland ( Bridgewater & Myers, 1979; Chadwick & Vasudev, 1989; Chadwick *et al.*, 1989; van Gool *et al.*, 2002; van Gool *et al.*, 2005). The belt consists dominantly of steeply-dipping, strike-slip (dominantly sinistral), E–W-striking shear zones, which are crosscut by gently dipping, top-SSW shear zones and nappe stacks, which formed in the Paleoproterozoic under greenschist facies conditions ( Bridgewater & Myers, 1979; Chadwick & Vasudev, 1989; Chadwick *et al.*, 1989; Kalsbeek *et al.*, 1993). The regional strike and kinematics of structures in the Ammassalik Belt are comparable to those of the WNW–ESE-striking thrust systems off the Faroe Islands ( Figure 2a–i, Figure 4a, and Figure 5a).

Smaller scale similarities include mylonitic fabrics (with sharp boundaries between intensely deformed and little deformed material) and imbricate structures, which are common both within the gently NNE-dipping shear zones of the Ammassalik Belt ( Bridgewater & Myers, 1979) and within the major thrust systems west and southwest of the Faroe Islands (e.g., asymmetric folds and duplexes separated by mylonitic thrust surfaces; Figure 2a–i). In addition, fold structures within shear zones in southeastern Greenland are of comparable size (hundred to a few hundreds of meters width and height) and geometry (south- to SSW-verging asymmetric, up to isoclinal; Chadwick & Vasudev, 1989, e.g., their figures 9 and 13) to those observed within the thrust systems in the study area ( Figure 2a–i). Moreover, the broad occurrences of sheared basic dykes within the shear zones at the Ammassalik Belt ( Bridgewater & Myers, 1979) may very well be present in the study area too and help enhance the acoustic impedance contrast allowing the imaging of the intra-thrust shear fabrics on the interpreted seismic reflection data (e.g., amplitude contrast between the mylonitic shear zones and asymmetric folds).

In northern Scotland, Paleoproterozoic structures in the Lewisian Complex include WNW–ESE-striking, amphibolite-facies, mylonitic shear zones formed during the Laxfordian Orogeny, such as the several kilometer-wide, > tens of kilometers long Laxford (or Tarbet), Canisp (or Stoer), Gairloch, Diabaig, Ness, and Langavat shear zones ( Attfield, 1987; Beach, 1974; Coward *et al.*, 1980; Coward & Park, 1987; Evans, 1965). These are also tightly folded into WNW–ESE-elongated, dome-shaped anticlines in the same way as the WNW–ESE-striking thrust systems on the Faroe Island continental shelf, which were reworked by NE–SW-striking Caledonian folds ( Figure 2a–i and Figure 4a). In addition, the double vergence of contractional structures off the Faroe Islands, i.e., (dominantly) top-SSW and (subsidiarily) top-NNE transport direction ( Figure 1b–c and Figure 2a–i), is consistent with the alternating top-SSW and top-NNE vergence of folds and shear zones onshore northern Scotland (e.g., top-NNE Laxford Shear Zone and top-SSW Diabaig Shear Zone; Figure 1c; Beach, 1974; Coward *et al.*, 1980; Coward & Park, 1987). Furthermore, some Laxfordian shear zones in northern Scotland also show top-SSW tectonic transport, e.g., Diabaig Shear Zone ( Beach, 1974; Coward *et al.*, 1980). Although most Laxfordian shear zones in northern Scotland display steep, subvertical geometries and evidence of (dominantly dextral) strike-slip movements ( Coward & Park, 1987), these may reflect Caledonian reactivation/overprinting due to NW-SE-oriented Caledonian contraction and/or portions of Paleoproterozoic shear zones that were folded during the Caledonian Orogeny similarly to the thrust systems off the Faroe Islands rather than exotic terranes or inliers (e.g., Friend *et al.*, 2008; Storey *et al.*, 2010; Figure 5b).

The strong similarities (e.g., strike, transport direction, fold and shear zone geometries, mylonitic fabrics) of structures in the Paleoproterozoic Ammassalik Belt in southeastern Greenland and coeval Laxfordian shear zones in northern Scotland with the mapped thrust systems off the Faroe Islands suggest that they are all part of the same (Laxfordian–Nagssugtoqidian) orogen. We therefore propose that the WNW–ESE-striking thrust systems west and southwest of the Faroe Islands formed during the late Paleoproterozoic ( Figure 5a).

Considering the occurrence of WNW–ESE-striking Inverian (ca. 2.49–2.48 Ga) shear zones in northern Scotland, which are crosscut by parallel Laxfordian shear zones (e.g., Attfield, 1987; Coward & Park, 1987), it is possible that the observed offshore thrust systems initiated during the Inverian Orogeny. However, more data and further work are needed to test this hypothesis. A late Paleoproterozoic age (ca. 1.8 Ga) for the presented thrust systems is therefore considered as a minimum age.

We note that the proposed relationship with late Paleoproterozoic Laxfordian–Nagssugtoqidian (and/or Inverian?) belts do not preclude a link of the mapped thrust systems with the Tornquist Zone and related fault segment, the Teisseyre–Tornquist Zone ( Krzywiec *et al.* 2022; Mazur *et al.*, 2015) and Sorgenfrei–Teisseyre Zone ( Phillips *et al.*, 2018; Ponikowska *et al.*, 2024). This would either mean that the Tornquist Zone formed along inherited late Paleoproterozoic thrusts, or that it had already been formed in the late Paleoproterozoic. The latter is possible considering the WNW–ESE strike of major late Paleoproterozoic Svecokarelian–Svecofennian structures in southern Baltica ( Bergh *et al.*, 2012; Nordgulen & Saintot, 2008; Saintot *et al.*, 2011), i.e., parallel to the Tornquist Zone ( Abramovitz *et al.*, 1999; Cotte *et al.*, 2002; Coward, 1990; Graversen, 2009; Hossein Shomali *et al.*, 2006; Narkiewicz *et al.*, 2015; Pegrum, 1984; Phillips *et al.*, 2018), and the proximity (< 100 km) of the southernmost Svecokarelian–Svecofennian structures in surface outcrops to the Sorgenfrei–Tornquist Zone in southeastern Sweden ( Saintot *et al.*, 2011). In addition, southern Baltica was also the locus of the late Paleoproterozoic Transscandinavian Igneous Belt, which also consists of WNW–ESE-striking structures in southernmost Sweden and Denmark, e.g., possibly late Paleoproterozoic WNW–ESE-striking gneissic fabrics on the island of Bornholm ( Johansson *et al.*, 2015), i.e., in the vicinity of the Sorgenfrei–Tornquist Zone.

***Late Neoproterozoic origin.*** A possible event is the late Neoproterozoic episode of deformation recorded by amphibolite-facies, top-west movements along the east-dipping Barnhill Shear Zone in northwestern Scotland ( Storey *et al.*, 2004) and in the Walls Metamorphic Series in the Shetland Islands ( Walker *et al.*, 2020). Although the N–S strike and top-west kinematics of the Barnhill Shear Zone differs from that of the major WNW–ESE-striking thrust systems west and southwest of the Faroe Islands, they may reflect folding of the shear zone during the Caledonian Orogeny as observed for the thrust systems in the study area ( Figure 2e, Figure 4a, and Figure 5b).

In eastern Europe, late Neoproterozoic deformation was recorded southwest of the WNW–ESE-striking Teisseyre–Tornquist Zone ( Belka *et al.*, 2003; Narkiewicz & Petecki, 2017; Zelazniewicz *et al.*, 2009). In addition, the imaginary prolongation of the Sorgenfrei–Tornquist Zone to the west-northwest lines up with the Wyville–Thomson fault zone ( Figure 1a–b). However, late Neoproterozoic deformation along the Teisseyre–Tornquist Zone is only preserved in exotic Gondwanan terranes in the southwest, and, thus far, the Tornquist Zone was only traced as far as the southern North Sea, where it controlled the formation and geometry of a series of WNW–ESE-striking, post-Caledonian rift basins (e.g., E–W-striking Farsund Basin; Figure 1b) and associated normal faults when it was reactivated as a major strike-slip fault ( Pegrum, 1984; Phillips *et al.*, 2018). More work is therefore needed to further examine this potential relationship.

Another fold-and-thrust belt that displays similar characteristics as those in the study area is the Timanian Orogen in northern Baltica. The Timanides are characterized by tens of km wide, tens of km thick, thousands of km long, dominantly top-SSW thrust systems, which extend from northwestern Russia to northern Norway, Svalbard and the western Barents Sea ( Koehl *et al.*, 2022; Koehl *et al.*, 2023a; Koehl *et al.*, 2023b; Koehl, 2024a; Koehl & Stokmo, 2024; Olovyanishnikov *et al.*, 2000; Siedlecka & Siedlecki, 1967). However, the Timanian Orogen is located some distance (c. one thousand km) from the study area.

Thus, although a late Neoproterozoic origin is possible for the interpreted thrust systems, a formation during the late Paleoproterozoic is more probable. It is therefore probable that the Barnhill Shear Zone in northern Scotland represents (or formed along) a folded portion of an inherited late Paleoproterozoic thrust system. This suggests that late Neoproterozoic deformation reactivated and/or overprinted preexisting late Paleoproterozoic orogens and related structures.

***Mid-Cenozoic origin.*** The positive relief at the location of major WNW–ESE- and NE–SW-striking macrofolds and thrust systems suggests recent activity along both structural trends. This is also suggested by the occurrence of multiple, high-angle, shallow (1.5–4.0 seconds TWT) reverse faults at the Wyville–Thomson and Ymir ridges ( Boldreel & Andersen, 1993 their figures 4 and 5, Boldreel & Andersen, 1998 their figures 4 and 5; Johnson *et al.*, 2005 their figure 6; Kimbell *et al*., 2016 their figure 2c; Stoker *et al.*, 2015; Jolley *et al.*, 2021; see Figure 1b–c for location). However, we did not find any convincing evidence of Cenozoic brittle reverse faulting within the Wyville–Thomson Ridge as suggested in Boldreel and Andersen ( 1993; 1998) and Kimbell *et al.* ( 2005; ramp-anticline complex).

The onlap of Cenozoic (Paleogene) volcanic lava flows and sedimentary rocks (e.g., Boldreel & Andersen, 1993 their figures 4 and 5, Boldreel & Andersen, 1998 their figures 4 and 5; Johnson *et al.*, 2005 their figure 6; Kimbell *et al.*, 2016 their figure 2c; Jolley *et al.*, 2021) onto metamorphosed and intensely folded basement rocks at depth (present study; Figure 3a–f) may suggest some contraction-related uplift during Cenozoic times. However, it must be noted that this may simply suggest the existence of paleotopography rather than contraction and uplift, and that lava flows do not behave like sediments and thickness variations and onlap features of lava flow successions therefore do not necessarily have the same geological implications as for sedimentary successions. Notably, the impact of existing topography on the thickness of lava flows significantly differs from that on sediments (e.g., Ganci *et al.*, 2018; Rizo, 2018; Richardson & Karlstrom, 2019). It is therefore possible that Paleogene lava flows in the study area mimic existing paleotopography onto which they were emplaced (e.g., lava flow sequence thickness variations in Jolley *et al.*, 2021).

In addition, the existence of topographic relief at present seafloor along both WNW–ESE- and NE–SW-striking macrofolds and thrust systems and the lack of ongoing tectonic contraction suggests that most (if not all) of present-day topography was partly inherited, possibly from the mid-Paleozoic Caledonian Orogeny (present study) and/or partly related to isostatic adjustments ( Smallwood, 2008). Magmatic-underplating-related uplift in the Faroe–UK region during the Cenozoic was ruled out ( Smallwood, 2008). Together with the absence of pervasive contractional deformation structures within Cenozoic volcanosedimentary successions (i.e., not as pervasive as suggested by Boldreel & Andersen, 1993; Boldreel & Andersen, 1998; Johnson *et al.*, 2005; Kimbell *et al.*, 2016; and Stoker *et al.*, 2015; Figure 2a–i), this indicates that Cenozoic contraction/transpression was, at most, mild if any. Thus, a Cenozoic origin for the observed WNW–ESE-striking thrust systems and related structures in metamorphosed basement rocks at depth (e.g., asymmetric folds, duplexes, mylonitic shear zones) can be ruled out ( Figure 2a–i and Figure 3a–f).

Nevertheless, it is possible that some Cenozoic contractional structures are present in the study area (e.g., Ymir Ridge; Boldreel & Andersen, 1993; Boldreel & Andersen, 1998; Johnson *et al.*, 2005; Kimbell *et al.*, 2016), the strike of which matches that of the interpreted WNW–ESE-striking thrust systems ( Figure 1b–c, Figure 2a–i, and Figure 4a), and that some of the topography at the structural highs in the study area is Cenozoic (e.g., Stoker *et al.*, 2005; Stoker *et al.*, 2013). Thus, the proposed late Paleoproterozoic thrust systems and their Caledonian overprints ( Figure 5a–b) may have been mildly reactivated/overprinted during Cenozoic contraction/transpression.

By contrast, there is no evidence for extensional reactivation at Wyville–Thomson Ridge ( Figure 2a–i). Hence, we may safely dismiss the presumed influence of an early Paleocene rifting event ( Ziska & Varming, 2008) in shaping the ridge and related structures, and an origin of the ridge along an inverted normal fault ( Boldreel & Andersen, 1993).

### Influence on NW–SE-striking post-Caledonian transfer zones and tectonosedimentary evolution

The interpreted WNW–ESE-striking thrust systems align with post-Caledonian transfer zones on the continental shelf, e.g., the Wyville–Thomson fault zone aligns with the Sula Transfer Zone in the southeast ( Bird *et al.*, 2016) and the southeastward prolongation of the Munkagrunnur fault zone coincides with the Judd Fault (also Judd Transfer Zone; Lamers & Carmichael, 1999; Stoker *et al.*, 2018; Sørensen, 2003; Figure 1c). The NW–SE-striking Sula transfer zone supposedly accommodates a switch of polarity of the main post-Caledonian, Devonian–Triassic normal faults, e.g., between the southeast-dipping Sula–Sgier Fault in the southwest and the northwest-dipping Shetland Spine Fault in the northeast ( Bird *et al.*, 2016; Figure 1c). Basement-seated thrust systems represent outstanding, (at least) hundreds of kilometers long, tens of kilometers wide, possibly tens of kilometers thick zones of weakness in the crust ( Figure 1a–c, Figure 2a–i, and Figure 4a) and it is therefore probable that they have had a considerable impact on the formation and evolution of subsequent structures. In the present case that is on accommodating switches of polarity of post-Caledonian faults as transfer zones ( Figure 5c). It is therefore proposed that the interpreted, WNW–ESE-striking, late Paleoproterozoic thrust systems continue southeast of the Faroe Islands, where they may be located at shallower crustal level and directly controlled the formation and evolution of the post-Caledonian transfer zones, as previously speculated by Kimbell *et al.* (2005). This is further supported by the occurrence of multiple NW–SE-striking transfer zones in northern Scotland (e.g., North Coast Transfer Zone), the Faroe Islands, and between the Faroe Islands and the Shetland Islands ( Bird *et al.*, 2016; Kimbell *et al.*, 2005; Lamers & Carmichael, 1999; Moy & Imber, 2009; Rumph *et al.*, 1993; Stoker *et al.*, 2018; Sørensen, 2003; Wilson *et al.*, 2010; Figure 1c), thus suggesting the existence of widespread WNW–ESE-striking fabrics in basement rocks in the region ( Figure 5c).

An interesting feature is the rectangular basin shape created by the NNE-dipping Judd Fault and Rona Fault at the southern edge of the Faroe–Shetland Basin. The former parallels the studied potential late Paleoproterozoic thrust systems, whereas the latter parallels Caledonian structures such as the Moine Thrust ( Figure 1b–c). It is therefore possible that the rectangular basin geometry is controlled by the two preexisting structural trends at depth.

Previous works have suggested various episodes of post-Caledonian uplift and erosion and rifting in the northeastern Atlantic region, including coeval contraction and extension at times (e.g., Dean *et al.*, 1999; Lamers & Carmichael, 1999; Stoker, 2016; Stoker *et al.*, 2017; Stoker *et al.*, 2018). It is of course possible that the late Paleoproterozoic thrust systems described herein were mildly reactivated and/or overprinted during these events. However, this is not clear from the data analyzed. As suggested by Dean *et al.* (1999) and Stoker (2016), a possible explanation is that the studied WNW–ESE-striking thrust systems were reactivated as strike-slip faults, which may explain the possibly occasionally coeval timing of contraction and extension along the margin. This would however be challenging to identify if the structures were only mildly reactivated/overprinted. Nevertheless, there should probably be clues such as high-angle to sub-vertical disruption surfaces reflecting truncation of the structures by recent strike-slip faults. This is not obvious from the data analyzed. More detailed work should therefore be performed in the Faroe–Shetland Basin area, where the relative abundance of exploration wells and seismic reflection data (including 3D data) would provide a more reliable tie between tectonic movements and sedimentary deposition along specific structures. A serious limitation might be the lack of deep seismic reflection data and poor seismic imaging below the Cenozoic volcanic successions in this area, thus impeding the correlation of shallow post-Caledonian brittle faults and sedimentary strata with deep, basement-seated structures.

The lack or mild character of post-Caledonian reactivation/overprinting of the studied WNW–ESE-striking late Paleoproterozoic thrust systems is in line with observations along the potentially related Tornquist Zone. For example, Phanerozoic sedimentary strata above the Teisseyre–Tornquist Zone are generally not disturbed by post-Caledonian tectonic events, dipping gently to the southwest ( Mazur *et al.*, 2015). Nonetheless, the Tornquist Zone and shows evidence of mild Late Cretaceous–early Cenozoic contraction (e.g., Ponikowska *et al.*, 2024), i.e., possibly similar to the study structures. Other potential episodes of mild reactivation along the Teisseyre–Tornquist Zone include Devonian–Mississippian extension, Variscan contraction in the Pennsylvanian, and Permian–Triassic extension ( Krzywiec *et al.*, 2022), the former and latter of which coincide with well-established extensional events in the study area off the Faroe Islands and northern Scotland.

## Implications for the Orogenic Bridge Theory and breakup in the northeastern Atlantic

The data suggest that late Paleoproterozoic thrust systems west of the Faroe Islands continue below the SDRs at the Iceland–Faroe Ridge, i.e., past the presumed continent–ocean transition ( Figure 2e). This suggests that the Iceland–Faroe Ridge consists (at least partly) of thinned, orogenic, continental crust ( Figure 2e and Figure 3d), thus supporting recent works (e.g., Foulger *et al.*, 2020; Foulger *et al.*, 2021).

The present results also support the Orogenic Bridge Theory, a recent concept which states that rifting style is controlled by preexisting orogens ( Koehl & Foulger, 2024). Rift-parallel orogens facilitate breakup, whereas rift-orthogonal orogens delay or impede breakup and localize the formation of major transform faults ( Koehl & Foulger, 2024). This concept and inconsistencies around the nature of the crust offshore ( Darbyshire *et al.*, 1998; Foulger, 2006; Foulger *et al.*, 2003; Foulger *et al.*, 2020; Foulger *et al.*, 2021; Menke *et al.*, 1995) were used to suggested the existence of elongate ribbons of (hyper-) extended orogenic crust between continents at the location of preexisting, rift-orthogonal orogens, e.g., at the late Paleoproterozoic Laxfordian–Nagssugtoqidian Orogen at the Greenland–Iceland–Faroe Ridge ( Koehl & Foulger, 2024). The formation of major transform faults along preexisting, rift-orthogonal, orogenic structures would explain the reworking of late Paleoproterozoic Laxfordian shear zones in northern Scotland into subvertical strike-slip structures ( Figure 5c).

The WNW–ESE-striking Laxfordian orogen (thickened continental crust) and related structures at the Faroe margin and their counterparts in southeastern Greenland were unsuitably oriented to accommodate thinning of the crust during rifting of the northeastern Atlantic because the main structures strike parallel to the extension direction. In addition, their low-angle geometry was unsuitable to accommodate transform faulting. Thus, a significant delay should be expected for continental breakup at the Greenland–Iceland–Faroe Ridge compared with adjacent areas along the rift axis.

**Figure 6.**
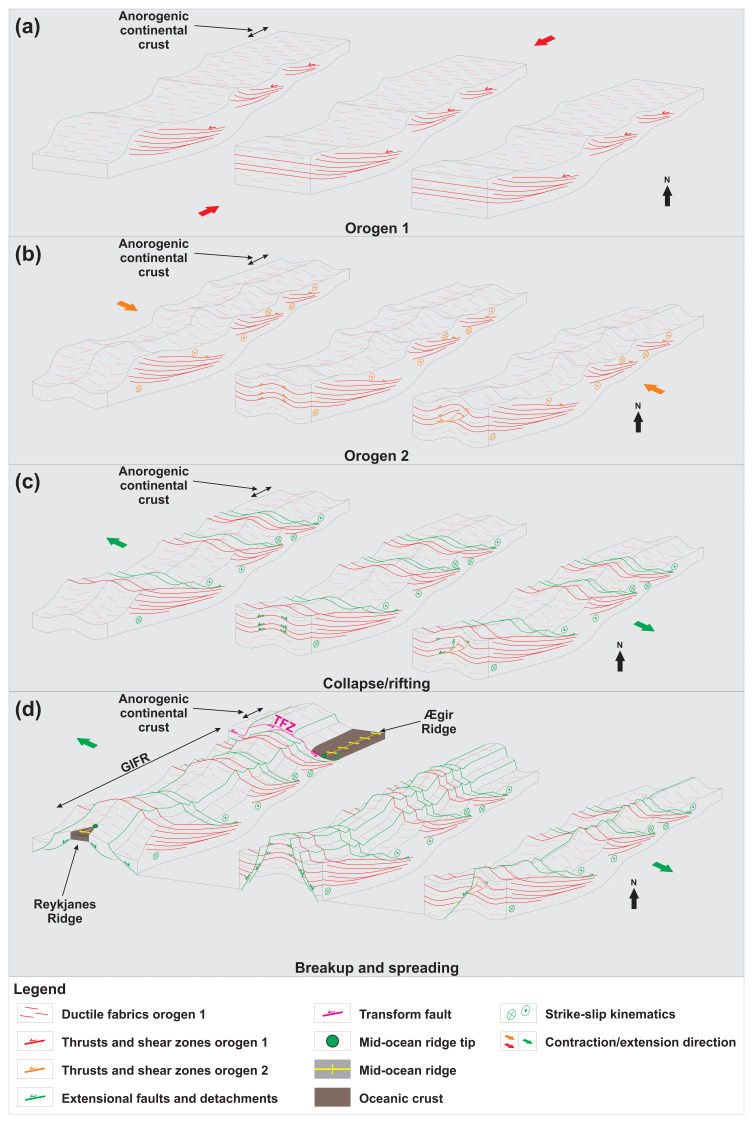
Model showing the development of an orogenic bridge at the Greenland–Iceland–Faroe Ridge through ( **a**– **b**) two orogeneses perpendicular to one another (( **a**) WNW–ESE-striking Laxofordian–Nagssugtoqidian Orogen and ( **b**) NE–SW-striking Caledonian Orogen), ( **c**) repeated extension along extensional faults and detachments and inherited orogenic structures, e.g., in the Devonian, Permian–Triassic, and Jurassic–Cretaceous, and ( **d**) breakup along inherited, reactivated and/or overprinted orogenic structures in the Cenozoic. Note that any number of additional orogeneses and/or extensional collapse/rifting may be added in between these events. Abbreviations: GIFR: Greenland–Iceland–Faroe Ridge; TFZ: Tjörnes fault zone.

Plume activity may very well be responsible for the intense magmatism recorded at the conjugate margin pair (southeastern Greenland and Faroe Islands) and in Iceland ( Dahl-Jensen *et al.*, 1997; Geoffroy *et al.*, 2022; Jolley *et al.*, 2021; Layfield *et al.*, 2023; Millett *et al.*, 2016; Walker *et al.*, 2022). However, should the studied late Paleoproterozoic structures extend farther offshore than what is presented in Figure 2e, the anomalously thick character of the crust under the Greenland–Iceland–Faroe Ridge may then be explained by the existence of a (continuous?) rift-orthogonal ribbon (i.e., continuous orogenic bridge; Koehl & Foulger, 2024; Figure 6a–d) or by several isolated blocks of continental crust entrapped by extensive magmatism (e.g., rifted oceanic magmatic plateau; Coffin & Eldholm, 1992; Rime *et al.*, 2024) or by several microcontinents similar to the Jan Mayen Microcontinent Complex and all rifted from one another and separated by regular, Penrose-like oceanic crust (e.g., Blischke *et al.*, 2016; Bott, 1985; Johnson & Heezen, 1967). Since the thickness of the Greenland–Iceland–Faroe Ridge is consistently higher than 20 km from southeastern Greenland to the Faroe shelf, a scenario with several microcontinents separated by Penrose-like oceanic crust is unlikely. Ongoing work using seismic reflection data within the Greenland–Iceland–Faroe Ridge suggests that the late Paleoproterozoic structures described in the present study extend at least to eastern Iceland ( Koehl *et al.*, 2025) and, thus, that the Greenland–Iceland–Faroe Ridge may be a continuous orogenic bridge ( Figure 6a–d; Koehl & Foulger, 2024), potentially developing into a rifted oceanic magmatic plateau (e.g., Rime *et al.*, 2024), i.e., the early phase towards its evolution to a ruptured orogenic bridge.

A continuous ribbon of continental crust between southeastern Greenland, Iceland, and the Faroe Islands is compatible with seafloor spreading at the Reykjanes Ridge and Kolbeinsey Ridge and with plume magmatism. The orogenic bridge may have formed during the late Paleoproterozoic Laxfordian–Nagssugtoqidian Orogeny ( Figure 6a) and was then reworked and tightened during NW–SE-oriented Caledonian contraction ( Figure 6b). The crust was then thinned during several ensuing episodes of extension, e.g., late–post-orogenic collapse in the Devonian and Permian–Triassic and Jurassic–Cretaceous rifting ( Figure 6c). While relatively thinner crust northeast and southwest of Iceland was broken up, highly thickened orogenic crust at the Greenland–Iceland–Faroe Ridge is still being thinned and stretching together with seafloor spreading at the adjacent Reykjanes and Kolbeinsey ridges ( Figure 6d; Koehl & Foulger, 2024).

This potentially has major implications for offshore areas where the nature of the crust is disputed, e.g., Mozambique Ridge ( König & Jokat, 2010; Ryzhova *et al.*, 2022), Madagascar Ridge ( Jacques *et al.*, 2019; O’Connor *et al.*, 2019; Sato *et al.*, 2022), Rio Grande Ridge ( Hoyer *et al.*, 2022; Ventura Santos *et al.*, 2019), Walvis Ridge ( Fromm *et al.*, 2017a; Fromm *et al.*, 2017b; Hoyer *et al.*, 2022), Mauritius Islands ( Torsvik *et al.*, 2013), and the Chagos–Laccadive Ridge ( Ajay *et al.*, 2010; Nair *et al.*, 2013). Notably, the present study shows a new way to map very old and deep orogenic systems using high-resolution seismic reflection data. The technique presented here may be used to track the offshore continuation of major preexisting orogenic structures into contested continental blocks, e.g., of the East African–Antarctica Orogen ( Abdelsalam *et al.*, 1998; Armistead, 2019; Bauer & Siemes, 2004; Boger *et al.*, 2015; Collins *et al.*, 2000; Collins *et al.*, 2012; De Waele *et al.*, 2011; Fritz *et al.*, 2013; Golynski & Jacobs, 2001; Hamimi *et al.*, 2022; Jacobs, 1999; Johnson, 2014; Key *et al.*, 1989; Mosley, 1993; Quick & Bosch, 1990; Ruppel *et al.*, 2015; Shackleton, 1996; Stern & Kröner, 1993) in the Madagascar and Mozambique ridges and Kuunga Orogen ( Axelsson *et al.*, 2020; Bingen *et al.*, 2009; Brandt *et al.*, 2014; Collins *et al.*, 2003; Dharmapriya *et al.*, 2015; Ghosh *et al.*, 2004; He *et al.*, 2018; Hirayama *et al.*, 2020; Sacchi *et al.*, 2000; Srinivasan & Rajeshdurai, 2010; Tucker *et al.*, 2007; Viola *et al.*, 2008) in the Rio Grande, Walvis, and Laccadive–Chagos ridges.

It has also potential applications for the definition of the term “terrane” and for the tectonics of presumed terranes in onshore areas, e.g., in the northern UK, which were thus far thought to have been separated by oceanic domains prior to being accreted during major orogenic events. For example, the Hebridean Craton in northern Scotland is believed to have been accreted to Avalonia during the Grampian and Caledonian orogenies ( Holdsworth *et al.*, 2012; Watson & Dunning, 1979). The possible relationship of the studied late Paleoproterozoic thrust systems with the Tornquist Zone challenges this paradigm by suggesting that the two cratons as well as Baltica may have been one and the same at least since the late Paleoproterozoic. The various terranes would thus reflect the strong heterogeneities in rock types and deformation intensity (including metamorphic grade and structure types) at various crustal levels within a single craton.

## Conclusions

1) The continental shelf off the Faroe Islands is crosscut by pre-Caledonian, tens of km wide, (at least) hundreds of km long, WNW–ESE-striking, dominantly top-SSW, pre-Caledonian thrust systems.
2) The dominant top-SSW kinematics of the WNW–ESE-striking thrust systems off the Faroe Islands suggest affinities with Paleoproterozoic orogens, e.g., the Ammassalik Belt in southeastern Greenland and the contemporaneous Svecokarelian–Svecofennian Orogen in Scandinavia, and with the Tornquist Zone in eastern Europe and the southern North Sea.
3) During the Caledonian Orogeny, the thrust systems were reworked into open, NE–SW-striking folds and controlled the formation of Caledonian thrusts and shear zones analogous to the Moine Thrust.
4) The late Paleoproterozoic thrust systems were possibly reactivated and/or overprinted during late Neoproterozoic and/or various episodes of post-Caledonian extension and contraction (transpression?). However, post-Caledonian reactivation/overprinting (if any) was of limited intensity.
5) The present work supports the Orogenic Bridge Theory by supporting the theory that the Greenland–Iceland–Faroe Ridge consists, at least partly, of thinned orogenic continental crust.

## Ethical approval and consent

Ethical approval and consent were not required.

## Data Availability

The data were provided by TGS ( https://www.tgs.com/ in the methods chapter of the manuscript). The data are private and subject to a data privacy agreement (cannot be shared, published, or showed without consent of TGS). Yet, TGS allowed us access to the data and gave us permission to publish the data (Academic License Agreement number NA0509-366). Any interested party may thus contact TGS directly with a research project proposal and be granted access to the data. Access to the data is free of charge for academic research purposes. DataverseNO: extended data for "Impact of sub-basalt thrust systems on the Faroe continental shelf for the late Paleoproterozoic–Cenozoic tectonic evolution of the margin", doi.org/10.18710/780M9P, 2024. The project contains the following extended data: -00_ReadMe.txt -Figure_2a.jpg -Figure_2b.jpg -Figure_2c.jpg -Figure_2d.jpg -Figure_2e.jpg -Figure_2f.jpg -Figure_2g.jpg -Figure_2h.jpg -Figure_2i.jpg The data are available under the terms of the Creative Commons Zero “No rights reserved” data waiver (CC0 1.0 Public domain dedication).
