# Supplementary material for: Impact of sub-basalt thrust systems on the Faroe continental shelf for the late Paleoproterozoic–Cenozoic tectonic evolution of the margin
Source: Open Res Eur. 2024 Dec 7;4:181. Originally published 2024 Aug 13. [Version 2] doi: 10.12688/openreseurope.18284.2 (PMC11809482; doi:10.12688/openreseurope.18284.2)
Supplement: Peer review history

# Reviewer report: Reviewer response for version 2

**DOI:** 10.21956/openreseurope.20606.r48255

**Published:** 2024-12-13

**Authors:** Martyn Stoker

**Affiliations:** 1 The University of Adelaide, Adelaide, South Australia, Australia

I am very satisfied with the author responses to my queries and suggestions. Thus, I am happy to approve the revised version 2 of the paper.

Is the study design appropriate and does the work have academic merit?

Yes

Is the work clearly and accurately presented and does it cite the current literature?

Yes

If applicable, is the statistical analysis and its interpretation appropriate?

Not applicable

Are all the source data underlying the results available to ensure full reproducibility?

Yes

Are the conclusions drawn adequately supported by the results?

Yes

Are sufficient details of methods and analysis provided to allow replication by others?

Yes

Reviewer Expertise:

Tectonostratigraphy of passive continental margins

I confirm that I have read this submission and believe that I have an appropriate level of expertise to confirm that it is of an acceptable scientific standard.

# Reviewer report: Reviewer response for version 2

**DOI:** 10.21956/openreseurope.20606.r48256

**Published:** 2024-12-13

**Authors:** Piotr Krzywiec

**Affiliations:** 1 Institute of Geological Sciences, Polish Academy of Sciences, Warsaw, Poland

Authors addressed all the issues I raised in my review and this paper might be, in my opinion, indexed in its final form. I still have however one last suggestion: information on the Teisseyre-Tornquist Zone and the Sorgenfrei-Tornquist Zone has been added but Authors decided to retain in their text also Tornquist Zone. I suggest to drop this term altogether, as such geological unit simply does not function in geological literature. TTZ and STZ are very different in terms of their crustal structure and geological history and they should not be treated as two segments of one, longer and broader, tectonic zone. Having only TTZ and STZ mentioned in the text would not hamper final impact of this paper; on the other hand, removing Tornquist Zone would result in clearer and more focused text that would be better understood by readers from this part of Europe.

Is the study design appropriate and does the work have academic merit?

Yes

Is the work clearly and accurately presented and does it cite the current literature?

Yes

If applicable, is the statistical analysis and its interpretation appropriate?

Not applicable

Are all the source data underlying the results available to ensure full reproducibility?

Partly

Are the conclusions drawn adequately supported by the results?

Partly

Are sufficient details of methods and analysis provided to allow replication by others?

Partly

Reviewer Expertise:

NA

I confirm that I have read this submission and believe that I have an appropriate level of expertise to confirm that it is of an acceptable scientific standard.

# Reviewer report: Reviewer response for version 1

**DOI:** 10.21956/openreseurope.19761.r43687

**Published:** 2024-11-14

**Authors:** Piotr Krzywiec

**Affiliations:** 1 Institute of Geological Sciences, Polish Academy of Sciences, Warsaw, Poland

This is interesting paper dealing with enigmatic structures within the basement of the Faroe–Shetland region.

 Authors used seismic data in order to propose system of compressional Precambrian deformation with some implications also for more recent, Phanerozoic history. Seismic imaging is, at such large depths and often beneath lava flows, not perfect and leaves quite a lot of room for speculations, but presented interpretation and models seem to viable.

 Paper is generally well written, properly illustrated, but there are some flaws (of different calibre) that require additional attention from the Authors:

 (1). Authors use term “Tornquist Zone” to describe zone of deformation known from S Scandinavia, Poland, and W Ukraine – however,  there is no single Tornquist Zone, instead two regional tectonic zones are distinguished: Teisseyre-Tornquist Zone in Poland and W Ukraine, and Sorgenfrei-Tornquist Zone in S Scandinavia (cf. Mazur et al., 2015, Is the Teisseyre-Tornquist Zone an ancient plate boundary of Baltica? Tectonics,  refer 1, and Ponikowska et al., 2024, Crustal‐Scale Pop‐Up Structure at the Junction of Two Continental‐Scale Deformation Zones in the Southern Baltic Sea, Tectonics, refer 2, for current views on TTZ and STZ).

 These zones have different crustal characteristics and different geological histories. Also, their location is different than what is shown on Fig. 1.

 Taking this into account, text on comparison of structures mapped in broad  Faroe–Shetland region and the “Tornquist Zone” needs to be modified, quite substantially in my opinion.

 (2) page 2: Authors claim that they used “newly established methodology” while in “Methods” chapter fairly standard workflow of  seismic reflection data interpretation is described, it would be good to explicitly describe new / novel aspect of seismic interpretation applied in this study

 (3) page 2: instead “strong seismic signal of the lavas” use “ strong attenuation of seismic energy by lavas” or something along those lines

 (3) link from age 6 https://doi.org/10.18710/780M9P does not work

 (4) page 16: Authors claim that  “ Structures in overlying post-Caledonian sedimentary and igneous rocks are irrelevant to the present study” - this is a bit puzzling as  in this paper there are numerous references to the post-Caledonian events, this should be rephrased and better clarified

 (5) page 16: it would be good to add figure with well tie instead of reference to other papers; also, location of wells used in this study should be shown on the map

 (6) page 22:  fairly well seismically documented example of post-Caledonian multiple reactivation along the TTZ could be found in Krzywiec et al. 2022, Together but separate: decoupled Variscan (late Carboniferous) and Alpine (Late Cretaceous–Paleogene) inversion tectonics in NW Poland, Solid Earth, refer 3

Is the study design appropriate and does the work have academic merit?

Yes

Is the work clearly and accurately presented and does it cite the current literature?

Yes

If applicable, is the statistical analysis and its interpretation appropriate?

Not applicable

Are all the source data underlying the results available to ensure full reproducibility?

Partly

Are the conclusions drawn adequately supported by the results?

Partly

Are sufficient details of methods and analysis provided to allow replication by others?

Partly

Reviewer Expertise:

NA

I confirm that I have read this submission and believe that I have an appropriate level of expertise to confirm that it is of an acceptable scientific standard, however I have significant reservations, as outlined above.

## References

[1] : Is the Teisseyre‐Tornquist Zone an ancient plate boundary of Baltica?. *Tectonics* .2015;34(12) : 10.1002/2015TC003934 2465-2477 10.1002/2015TC003934

[2] : Crustal‐Scale Pop‐Up Structure at the Junction of Two Continental‐Scale Deformation Zones in the Southern Baltic Sea. *Tectonics* .2024;43(4) : 10.1029/2023TC008066 10.1029/2023TC008066

[3] : Together but separate: decoupled Variscan (late Carboniferous) and Alpine (Late Cretaceous–Paleogene) inversion tectonics in NW Poland. *Solid Earth* .2022;13(3) : 10.5194/se-13-639-2022 639-658 10.5194/se-13-639-2022

# Response

**Published:** 2024-11-26

**Authors:** Jean-Baptiste Koehl

**Affiliations:** Geosciences, Universitetet i Oslo, Oslo, Oslo, Norway

Dear Prof. Krzywiec, thank you very much for your input on the manuscript, it is highly appreciated. Here is our reply to your comments. We hope the changes we implemented improve the shortcomings of the manuscript highlighted by your comments and suggestions. Please do not hesitate to contact us shall this not be the case for some comments. **Comments by the reviewer**

Comment 1: (1). Authors use term “Tornquist Zone” to describe zone of deformation known from S Scandinavia, Poland, and W Ukraine – however,  there is no single Tornquist Zone, instead two regional tectonic zones are distinguished: Teisseyre-Tornquist Zone in Poland and W Ukraine, and SorgenfreiTornquist Zone in S Scandinavia (cf. Mazur et al., 2015, Is the Teisseyre-Tornquist Zone an ancient plate boundary of Baltica? Tectonics,  refer 1, and Ponikowska et al., 2024, Crustal‐Scale Pop‐Up Structure at the Junction of Two Continental‐Scale Deformation Zones in the Southern Baltic Sea, Tectonics, refer 2, for current views on TTZ and STZ). These zones have different crustal characteristics and different geological histories. Also, their location is different than what is shown on Fig. 1. Taking this into account, text on comparison of structures mapped in broad  Faroe–Shetland region and the “Tornquist Zone” needs to be modified, quite substantially in my opinion.

Response: Agreed. The authors of the present manuscript concede that the location, geometry, and name of the various segments of the Tornquist Zone are not are accurate as they should be. Some specific details on the tectonic evolution of the Tornquist Zone and its various segments are indeed relevant to include to the present manuscript and the authors of the present manuscript thank the reviewer for calling the attention to the work by Mazur et al. (2015), Krzywiec et al. (2022), and Ponikowska et al. (2024). The authors of the present manuscript also concede that a scenario in which a post-Paleoproterozoic Tornquist Zone is controlled by late Paleoproterozoic fabrics and structures should be discussed in the present manuscript. Changes: Redesigned Figure 1a–b with the correct location and geometry of the various segments of the Tornquist Zone, the Sorgenfrei–Tornquist and Teisseyre–Tornquist zones. In addition, added “The geometry and location of the Sorgenfrei–Tornquist Zone and Teisseyre–Tornquist Zone are from Phillips *et al.* (2018), Krzywiec *et al.* (2022), and Ponikowska *et al.* (2024).” and related abbreviations to the caption of Figure 1b. Also separated the abbreviations for Figure 1b and Figure 1c in the figure caption. Rewrote the last two sentences of the Abstract into “The thrust systems also align with the Tornquist Zone in eastern Europe and the North Sea, thus suggesting either that they controlled the formation of the Tornquist Zone or a possibly much longer (Paleoproterozoic?) tectonic history for the Tornquist Zone. Conclusions The Faroe Island margin is crosscut by late Paleoproterozoic Laxfordian–Nagssugtoqidian thrust systems, which controlled further tectonic development of the margin” to account for the case scenario in which the Tornquist Zone formed along preexisting Paleoproterozoic thrust systems. Rewrote the sixth paragraph in the “Late Paleoproterozoic origin” sub-section into “We note that the proposed relationship with late Paleoproterozoic Laxfordian–Nagssugtoqidian (and/or Inverian?) belts do not preclude a link of the mapped thrust systems with the Tornquist Zone and related fault segment, the Teisseyre–Tornquist Zone ( Mazur *et al.*, 2015; Krzywiec *et al.* 2022) and Sorgenfrei–Teisseyre Zone ( Phillips *et al.*, 2018; Ponikowska *et al.*, 2024). This would either mean that the Tornquist Zone formed along inherited late Paleoproterozoic thrusts, or that it had already been formed in the late Paleoproterozoic. The latter is possible considering the WNW–ESE strike of major late Paleoproterozoic Svecokarelian–Svecofennian structures in southern Baltica ( Bergh *et al.*, 2012; Nordgulen & Saintot, 2008; Saintot *et al.*, 2011), i.e., parallel to the Tornquist Zone ( Abramovitz *et al.*, 1999; Cotte *et al.*, 2002; Coward, 1990; Graversen, 2009; Hossein Shomali *et al.*, 2006; Narkiewicz *et al.*, 2015; Pegrum, 1984; Phillips *et al.*, 2018), and the proximity (< 100 km) of the southernmost Svecokarelian–Svecofennian structures in surface outcrops to the Sorgenfrei–Tornquist Zone in southeastern Sweden ( Saintot *et al.*, 2011). In addition, southern Baltica was also the locus of the late Paleoproterozoic Transscandinavian Igneous Belt, which also consists of WNW–ESE-striking structures in southernmost Sweden and Denmark, e.g., possibly late Paleoproterozoic WNW–ESE-striking gneissic fabrics on the island of Bornholm ( Johansson *et al.*, 2015), i.e., in the vicinity of the Sorgenfrei–Tornquist Zone.” and the second paragraph in the “Late Neoproterozoic origin” sub-section into “In eastern Europe, late Neoproterozoic deformation was recorded southwest of the WNW–ESE-striking Teisseyre–Tornquist Zone ( Belka *et al.*, 2003; Narkiewicz & Petecki, 2017; Zelazniewicz *et al.* 2009). In addition, the imaginary prolongation of the Sorgenfrei–Tornquist Zone to the west-northwest lines up with the Wyville–Thomson fault zone ( Figure 1a–b). However, late Neoproterozoic deformation along the Teisseyre–Tornquist Zone is only preserved in exotic Gondwanan terranes in the southwest, and, thus far, the Tornquist Zone was only traced as far as the southern North Sea, where it controlled the formation and geometry of a series of WNW–ESE-striking, post-Caledonian rift basins (e.g., E–W-striking Farsund Basin; Figure 1b) and associated normal faults when it was reactivated as a major strike-slip fault ( Pegrum, 1984; Phillips *et al.*, 2018). More work is therefore needed to further examine this potential relationship.”. Also added reference to Mazur et al. (2015), Krzywiec et al. (2022), and Ponikowska et al. (2024) to the reference list. Finally, added another paragraph at the end of the “Influence on NW–SE-striking post-Caledonian transfer zones and tectonosedimentary evolution” section: “The lack or mild character of post-Caledonian reactivation/overprinting of the studied WNW–ESE-striking late Paleoproterozoic thrust systems is in line with observations along the potentially related Tornquist Zone. For example, Phanerozoic sedimentary strata above the Teisseyre–Tornquist Zone are generally not disturbed by post-Caledonian tectonic events, dipping gently to the southwest ( Mazur *et al.*, 2015). Nonetheless, the Tornquist Zone and shows evidence of mild Late Cretaceous–early Cenozoic contraction (e.g., Ponikowska *etal.*, 2024), i.e., possibly similar to the study structures.” to further illustrate the resemblance of the tectonic evolution of the Tornquist Zone and the studied structures off the Faroe Islands.  

Comment 2: (2) page 2: Authors claim that they used “newly established methodology” while in “Methods” chapter fairly standard workflow of  seismic reflection data interpretation is described, it would be good to explicitly describe new / novel aspect of seismic interpretation applied in this study.

Response: Agreed. The sentence is poorly phrased and this is not a new methodology and the newly established facies/character of major, mylonitic thrust systems on seismic reflection data. Changes: Rewrote the end of the sentence into “using the newly established seismic facies of major thrust systems”. Also shortened the abstract to 300 words.  

Comment 3: (3) page 2: instead “strong seismic signal of the lavas” use “ strong attenuation of seismic energy by lavas” or something along those lines.

Response: Agreed. Changes: Rewrote the targeted phrase in the plain language summary into “strong attenuation of seismic energy by lavas”.  

Comment 4: (3) link from age 6 https://doi.org/10.18710/780M9P does not work.

Response: Agreed. The main author of the present manuscript presents his apologies to the reviewer. The dataset, though ready to be published, was never submitted. Changes: Submitted the dataset to the repository. It should now be accessible.  

Comment 5: (4) page 16: Authors claim that  “ Structures in overlying post-Caledonian sedimentary and igneous rocks are irrelevant to the present study” - this is a bit puzzling as  in this paper there are numerous references to the post-Caledonian events, this should be rephrased and better clarified.

Response: Agreed. The sentence is poorly phrased. The authors of the present manuscript simply wanted to emphasize that the main structures investigated in the present study are pre-Caledonian (possibly late Paleoproterozoic) and Caledonian structures and were only mildly (if at all) reworked during post-Caledonian events. Post-Caledonian structures and tectonism therefore have little implications for the geometry of the studied structures. The authors of the present manuscript concede that this should be better specified. Changes: The paragraph was rewritten into “Structures in overlying post-Caledonian sedimentary and igneous rocks only mildly (if at all) rework the studied pre-Cretaceous structures and, thus, have little impact on the geometry of the studied structures. Post-Caledonian structures were therefore not investigated, except where they showed a clear relationship with the studied basement-seated structures (e.g., merging, truncating, and/or reworking).”.  

Comment 6: (5) page 16: it would be good to add figure with well tie instead of reference to other papers; also, location of wells used in this study should be shown on the map Response: Partly agreed. The well to which the interpretation was tied is displayed in Figure 1c. This was not specified in the text. However, the well tie was performed and published in a previous study (Jolley et al., 2021). The seismic reflection data used to tie to data are not part of the present study and require other permission/license, which are not covered by the present manuscript. Thus, the well tie and related seismic reflection data cannot be added to the present study. They are nevertheless available in figure 19 in Jolley et al. (2021). Changes: Added “( Figure 1c) in the fourth paragraph in the Method chapter.  

Comment 7: (6) page 22:  fairly well seismically documented example of post-Caledonian multiple reactivation along the TTZ could be found in Krzywiec et al. 2022, Together but separate: decoupled Variscan (late Carboniferous) and Alpine (Late Cretaceous–Paleogene) inversion tectonics in NW Poland, Solid Earth, refer 3.

Response: Agreed.

 See response to comment 1. Changes: See response to comment 1. Also added the following sentence at the end of the newly added paragraph in the “Influence on NW–SE-striking post-Caledonian transfer zones and tectonosedimentary evolution” section: “Other potential episodes of mild reactivation along the Teisseyre–Tornquist Zone include Devonian–Mississippian extension, Variscan contraction in the Pennsylvanian, and Permian–Triassic extension (Krzywiec et al., 2022), the former and latter of which coincide with well-established extensional events in the study area off the Faroe Islands and northern Scotland.” to further specify potential resemblance in the tectonic evolution of the Faroe Islands and northern Scotland region with the Tornquist Zone in eastern Europe.

# Reviewer report: Reviewer response for version 1

**DOI:** 10.21956/openreseurope.19761.r43656

**Published:** 2024-09-04

**Authors:** Martyn Stoker

**Affiliations:** 1 The University of Adelaide, Adelaide, South Australia, Australia

**Review of Koehl et al. – Impact of sub-basalt thrust systems on the Faroe continental shelf for the late Paleoproterozoic–Cenozoic tectonic evolution of the margin**

 It has long been considered that the cross-cutting structural grain of the pre-Cenozoic basement of the Faroe–Shetland region reflects the interaction of long-lived NE- (Caledonian)and NW-trending (Precambrian) lineaments, which were periodically and variably reactivated throughout the breakup of Pangaea (cf. Ritchie et al. 2011 and references therein). However, this new article by Koehl et al. identifies and describes the character of these features – interpreted as late Paleoproterozoic margin-orthogonal thrust systems – as observed on seismic reflection data, arguably for the first time in this region, enabling a potential insight into the evolution of this structural framework. 

 The paper is very well written and presented, with the structural analysis greatly benefitting from the availability of reprocessed seismic reflection data. This allowed for the interpretation of the seismic character of the deep basement of the Faroe–Shetland region, including beneath the thick Cenozoic lava flows that characterise the Faroe Islands continental margin. Although seismic interpretation always carries a degree of ambiguity, the structural interpretation in this paper is backed up by a significant knowledge of the internal geometry of thrust systems thereby enabling the potential seismic recognition of key criteria, such as mylonitic shear surfaces, asymmetric folds and their vergence direction, duplexes, and minor brittle thrusts. Thus, at the very least, the paper provides an important model that should be considered and tested in any future studies in this region. 

**Comments**

 My main experience in the Faroe–Shetland region concerns its late Palaeozoic–Cenozoic tectonostratigraphic development (Stoker *et al*. 2017). Based on this, I have a couple of general comments for the authors to think about: 

 1.    Whereas the authors’ state (page 16, para 2) that ‘Structures in the … post-Caledonian sedimentary and igneous rocks are irrelevant to the present study…’, I would argue that the post-Caledonian rock record in the Faroe–Shetland region is  entirely relevant when considering the formation/longevity of the basement structural system. Key observations from my studies include:

- It is well established that the eastern and southern margin of the Faroe-Shetland Basin and its component sub-basins have been controlled by the NE-trending Rona Fault and the WNW-trending Judd Fault throughout its development (Lamers & Carmichael 1999; Stoker *et al*. 2018). 
- This basin configuration and its infill has been developing since at least the Jurassic (and possibly the Permo-Triassic) (Ritchie *et al*. 2011; Stoker *et al*. 2017). 
- The Cretaceous stratigraphic record of the Faroe-Shetland Basin reveals a sedimentary succession that is punctuated by episodes of uplift, erosion and contractional deformation that can be linked to a pattern of coeval extension and compression consistent with intra-plate strike-slip tectonism (Dean *et al. *1999; Stoker 2016). 
- This tectonic instability continued throughout the Paleocene and Eocene development of the basin, spanning the breakup of the SE Greenland and Faroe–Shetland conjugate margins (Stoker *et al*. 2018). 
- The structural disposition of the Eocene sediments and the onlap pattern of the overlying Oligocene–Neogene succession indicates that the present-day deep-water bathymetry of the Faroe–Shetland region most probably developed in the late Palaeogene–Early Neogene interval (Stoker *et al*. 2005, 2013). Whether this is a result of contraction or loss of dynamic support following plate breakup remains unclear. 

This history of basin development and sedimentation preserved in the Faroe-Shetland Basin is a clear record of episodic reactivation throughout the Mesozoic and Cenozoic of an existing underlying structural framework. Whereas I have offered explanations for the above observations in my own publications, I am intrigued to wonder how they might relate to this new model. Thus, I would ask the authors to consider the post-Caledonian stratigraphic record as one way of testing their model. Based on this tectonostratigraphic framework, it is obvious that any Mid-Cenozoic contraction that might be linked to their WNW–ESE-striking thrust system is just a response to episodic reactivation (as concluded in the paper). Perhaps the Discussion could be modified so that a subsection on 'post-Caledonian reactivation' highlights the significance of the NE- and NW-trending lineaments to tectonostratigraphic development. The current 'Mid-Cenozoic origin' text (page 21) might also form part of this subsection. Having said that, the abundance of inversion domes generated and episodically growing throughout the Cenozoic (Johnson *et al*. 2005; Ritchie *et al*. 2008) is noteworthy and clearly reflects a protracted phase of contraction/transpression either side of plate breakup. Perhaps this suggests that Cenozoic compression in the Faroe–Shetland region is more related to oceanic spreading. Any thoughts?

 2.    It would be useful to have a paragraph that considers the general implications of this model for the evolution of the NE Atlantic region in general, especially regarding plate breakup. Significantly, the authors mention that the Greenland-Iceland-Faroe Ridge partly consists of continental crust, which supports the Orogenic Bridge Theory.  Thus, what impact does this model have on plate breakup in the NE Atlantic? What bearing (if any) does it have on the ‘plates vs. plumes’ controversy, which has been going on for several decades.

*One minor detail:* in the plain language summary, on the fourth line from the bottom, ‘1.8 million years’ should read ‘1.8 billion years’.

**Actions**

 I leave it up to the authors to consider whether or not to act on my suggestions. However, I do feel that their assessment that the post-Caledonian tectonostratigraphic framework is 'irrelevant' to this study leaves the Discussion unbalanced and lacking crucial information that has an important bearing on the 'Timing of Formation...' and the ensuing history of reactivation.

 Nevertheless, I think that this is a very good paper with exciting consequences for better understanding the structural framework of the Faroe-Shetland continental margin.

 Martyn Stoker

 03-September-2024

Is the study design appropriate and does the work have academic merit?

Yes

Is the work clearly and accurately presented and does it cite the current literature?

Yes

If applicable, is the statistical analysis and its interpretation appropriate?

Not applicable

Are all the source data underlying the results available to ensure full reproducibility?

Yes

Are the conclusions drawn adequately supported by the results?

Yes

Are sufficient details of methods and analysis provided to allow replication by others?

Yes

Reviewer Expertise:

Tectonostratigraphy of passive continental margins

I confirm that I have read this submission and believe that I have an appropriate level of expertise to confirm that it is of an acceptable scientific standard, however I have significant reservations, as outlined above.

## References

[1] : Rifting and the development of the Faeroe-Shetland Basin. *Geological Society, London, Petroleum Geology Conference Series* .1999;5(1) : 10.1144/0050533 533-544 10.1144/0050533

[2] : Aspects of the Cenozoic deformational history of the Northeast Faroe–Shetland Basin, Wyville–Thomson Ridge and Hatton Bank areas. *Geological Society, London, Petroleum Geology Conference Series* .2005;6(1) : 10.1144/0060993 993-1007 10.1144/0060993

[3] : The Paleocene deepwater sandstone play West of Shetland. *Geological Society, London, Petroleum Geology Conference Series* .1999;5(1) : 10.1144/0050645 645-659 10.1144/0050645

[4] : The effects of Cenozoic compression within the Faroe-Shetland Basin and adjacent areas. *Geological Society, London, Special Publications* .2008;306(1) : 10.1144/SP306.5 121-136 10.1144/SP306.5

[5] : Geology of the Faroe–Shetland Basin and adjacent areas. British Geological Survey Research Report. *British Geological Survey Jarðfeingin* .2011; Reference source

[6] : Cretaceous tectonostratigraphy of the Faroe–Shetland region. *Scottish Journal of Geology* .2016;52(1) : 10.1144/sjg2016-004 19-41 10.1144/sjg2016-004

[7] : A record of Eocene (Stronsay Group) sedimentation in BGS borehole 99/3, offshore NW Britain: Implications for early post-break-up development of the Faroe–Shetland Basin. *Scottish Journal of Geology* .2013;49(2) : 10.1144/sjg2013-001 133-148 10.1144/sjg2013-001

[8] : A rift-to-drift record of vertical crustal motions in the Faroe–Shetland Basin, NW European margin: establishing constraints on NE Atlantic evolution. *Journal of the Geological Society* .2018;175(2) : 10.1144/jgs2017-076 263-274 10.1144/jgs2017-076

[9] : Sedimentary and oceanographic responses to early Neogene compression on the NW European margin. *Marine and Petroleum Geology* .2005;22(9-10) : 10.1016/j.marpetgeo.2005.01.009 1031-1044 10.1016/j.marpetgeo.2005.01.009

[10] : An overview of the Upper Palaeozoic–Mesozoic stratigraphy of the NE Atlantic region. *Geological Society, London, Special Publications* .2017;447(1) : 10.1144/SP447.2 11-68 10.1144/SP447.2

# Response

**Published:** 2024-11-26

**Authors:** Jean-Baptiste Koehl

**Affiliations:** Geosciences, Universitetet i Oslo, Oslo, Oslo, Norway

Dear Prof. Stoker, thank you very much for your input on the manuscript, it is highly appreciated. Here is our reply to your comments. We hope the changes we implemented improve the shortcomings of the manuscript highlighted by your comments and suggestions. Please do not hesitate to contact us shall this not be the case for some comments.

**Comments by the reviewer**

**Comment 1: 1.** Whereas the authors’ state (page 16, para 2) that ‘Structures in the … post-Caledonian sedimentary and igneous rocks are irrelevant to the present study…’, I would argue that the post Caledonian rock record in the Faroe–Shetland region is  entirely relevant when considering the formation/longevity of the basement structural system.

**Response**: Agreed. The sentence is poorly phrased. The authors of the present manuscript simply wanted to emphasize that the main structures investigated in the present study are pre-Caledonian (possibly late Paleoproterozoic) and Caledonian structures and were only mildly (if at all) reworked during post-Caledonian events. Post-Caledonian structures and tectonism therefore have little implications for the geometry of the studied structures. The authors of the present manuscript concede that this should be better specified.

Changes: The paragraph was rewritten into “Structures in overlying post-Caledonian sedimentary and igneous rocks only mildly (if at all) rework the studied pre-Cretaceous structures and, thus, have little impact on the geometry of the studied structures. Post-Caledonian structures were therefore not investigated, except where they showed a clear relationship with the studied basement-seated structures (e.g., merging, truncating, and/or reworking).”.  

**Comment 2:** Key observations from my studies include: It is well established that the eastern and southern margin of the Faroe-Shetland Basin and its component sub-basins have been controlled by the NE-trending Rona Fault and the WNW-trending Judd Fault throughout its development (Lamers & Carmichael 1999; Stoker et al. 2018).

**Response:** Agreed. The reviewer makes a compelling point about the WNW–ESE-striking Judd Fault, which coincides with the southeastward continuation of the Munkargrunnur fault zone.

Changes: Modified the title of the “Influence on NW–SE-striking post-Caledonian transfer zones” into “Influence on NW–SE-striking post-Caledonian transfer zones and tectonosedimentary evolution”. Added the location of the Judd Transfer Zone in Figure 1b–c and related abbreviation to the figure caption. Added “) and the southeastward prolongation of the Munkagrunnur fault zone coincides with the Judd Fault (also Judd Transfer Zone; Lamers & Carmichael, 1999; Stoker *et al.*, 2018; Sørensen, 2003;” section and reference to Lamers and Carmichael (1999), Stoker *et al.* (2018), Sørensen (2003) later in the same paragraph. Also added one paragraph after the first paragraph “An interesting feature is the rectangular basin shape created by the NNE-dipping Judd Fault and Rona Fault at the southern edge of the Faroe–Shetland Basin. The former parallels the studied potential late Paleoproterozoic thrust systems, whereas the latter parallels Caledonian structures such as the Moine Thrust ( Figure 1b–c). It is therefore possible that the rectangular basin geometry is controlled by the two preexisting structural trends at depth.”.  

**Comment 3:** This basin configuration and its infill has been developing since at least the Jurassic (and possibly the Permo-Triassic) (Ritchie et al. 2011; Stoker et al. 2017).

**Response:** The various episodes of post-Caledonian uplift and erosion and rifting suggested in Stoker et al. (2017) may of course have mildly reactivated the studied structures. The authors of the present manuscript concede that this should be better reflected in the present manuscript.

Changes: Added mention of Permian–Triassic and Jurassic–Cretaceous rifting in the “Implications for the Orogenic Bridge Theory and breakup of the northeastern Atlantic” section and the new Figure 6c and a third paragraph to the “Influence on NW–SE-striking post-Caledonian transfer zones and tectonosedimentary evolution” section: “Previous works have suggested various episodes of post-Caledonian uplift and erosion and rifting in the northeastern Atlantic region, including coeval contraction and extension at times (e.g., Dean *et al.*, 1999; Lamers & Carmichael, 1999; Stoker, 2016; Stoker et al., 2017, 2018). It is of course possible that the late Paleoproterozoic thrust systems described herein were mildly reactivated and/or overprinted during these events. However, this is not clear from the data analyzed. As suggested by Dean et al. (1999) and Stoker (2016), a possible explanation is that the studied WNW–ESE-striking thrust systems were reactivated as strike-slip faults, which may explain the possibly occasionally coeval timing of contraction and extension along the margin. This would however be challenging to identify if the structures were only mildly reactivated/overprinted. Nevertheless, there should probably be clues such as high-angle to sub-vertical disruption surfaces reflecting truncation of the structures by recent strike-slip faults. This is locally the case in shallow sedimentary strata, e.g., at the Ymir Ridge (Boldreel and Andersen, 1993 their figures 4 and 5, Boldreel and Andersen, 1998 their figures 4 and 5; Johnson et al., 2005 their figure 6; Kimbell et al., 2016 their figure 2c; Stoker et al., 2015; Jolley et al., 2021). However, it is not obvious within pre-Cretaceous basement rocks from the data analyzed. More detailed work should therefore be performed in the Faroe–Shetland Basin area, where the relative abundance of exploration wells and seismic reflection data (including 3D data) would provide a more reliable tie between tectonic movements and sedimentary deposition along specific structures. A serious limitation might be the lack of deep seismic reflection data and poor seismic imaging below the Cenozoic volcanic successions in this area, thus impeding the correlation of shallow post-Caledonian brittle faults and sedimentary strata with deep, basement-seated structures.”  

**Comment 4:** The Cretaceous stratigraphic record of the Faroe-Shetland Basin reveals a sedimentary succession that is punctuated by episodes of uplift, erosion and contractional deformation that can be linked to a pattern of coeval extension and compression consistent with intraplate strike-slip tectonism (Dean et al. 1999; Stoker 2016).

**Response**: Agreed. See response to comment 3.

Changes: See response to comment 3.  

**Comment 5:** This tectonic instability continued throughout the Paleocene and Eocene development of the basin, spanning the breakup of the SE Greenland and Faroe–Shetland conjugate margins (Stoker et al. 2018).

**Response:** See response to comment 3.

Changes: See response to comment 3.  

**Comment 6:** The structural disposition of the Eocene sediments and the onlap pattern of the overlying Oligocene–Neogene succession indicates that the present-day deep-water bathymetry of the Faroe–Shetland region most probably developed in the late Palaeogene–Early Neogene interval (Stoker et al. 2005, 2013). Whether this is a result of contraction or loss of dynamic support following plate breakup remains unclear.

**Response:** Agreed. The authors of the present manuscript thank the reviewer for recommending valuable previous works on the tectonic evolution of the margin to include to the present study.

Changes: Added reference to Stoker et al. (2005, 2013) in the fourth paragraph of the “Mid-Cenozoic origin” sub-section and to the reference list.  

**Comment 7:** This history of basin development and sedimentation preserved in the Faroe-Shetland Basin is a clear record of episodic reactivation throughout the Mesozoic and Cenozoic of an existing underlying structural framework. Whereas I have offered explanations for the above observations in my own publications, I am intrigued to wonder how they might relate to this new model. Thus, I would ask the authors to consider the post-Caledonian stratigraphic record as one way of testing their model. Based on this tectonostratigraphic framework, it is obvious that any Mid-Cenozoic contraction that might be linked to their WNW–ESE-striking thrust system is just a response to episodic reactivation (as concluded in the paper). Perhaps the Discussion could be modified so that a subsection on 'post-Caledonian reactivation' highlights the significance of the NE- and NW-trending lineaments to tectonostratigraphic development. The current 'Mid-Cenozoic origin' text (page 21) might also form part of this subsection. Having said that, the abundance of inversion domes generated and episodically growing throughout the Cenozoic (Johnson et al. 2005; Ritchie et al. 2008) is noteworthy and clearly reflects a protracted phase of contraction/transpression either side of plate breakup. Perhaps this suggests that Cenozoic compression in the Faroe–Shetland region is more related to oceanic spreading. Any thoughts?

**Response:** Agreed. The manuscript does need to further expand on the potential impact of the studied late Paleoproterozoic structures on the post-Caledonian evolution of the margin. See responses to comments 1 to 6. The reviewer also raises a highly relevant question regarding the cause of Cenozoic contraction around the Faroe Islands. Considering the potential continental nature of the crust at the Greenland–Iceland–Faroe Ridge, then Cenozoic contraction may be related to extension of the orogenic crust there and to seafloor spreading at the Reykjanes and Kolbeinsey ridges. However, as mentioned in the present study, the phases of tectonism that contributed the most to shape the studied structures are the late Paleoproterozoic Laxfordian–Nagssugtoqidian Orogeny and mid-Paleozoic Caledonian Orogeny. Subsequent tectonic events had only a limited impact on these structures, if any at all. Thus, it is probable that most of the relief created by the studied structures was already established in the mid-Paleozoic.

Changes: See also responses to comments 1 to 6. Also added “possibly from the mid-Paleozoic Caledonian Orogeny” in the third paragraph in the “Mid-Cenozoic origin” sub-section, and rewrote the fourth point of the conclusion into “The late Paleoproterozoic thrust systems were possibly reactivated and/or overprinted during late Neoproterozoic and/or various episodes of post-Caledonian extension and contraction (transpression?). However, post-Caledonian reactivation/overprinting (if any) was of limited intensity.”.  

**Comment 8: 2**.    It would be useful to have a paragraph that considers the general implications of this model for the evolution of the NE Atlantic region in general, especially regarding plate breakup. Significantly, the authors mention that the Greenland-Iceland-Faroe Ridge partly consists of continental crust, which supports the Orogenic Bridge Theory.  Thus, what impact does this model have on plate breakup in the NE Atlantic? What bearing (if any) does it have on the ‘plates vs. plumes’ controversy, which has been going on for several decades.

**Response:** Agreed. This is an excellent suggestion by the reviewer.

Changes: Added “The WNW–ESE-striking Laxfordian orogen (thickened continental crust) and related structures at the Faroe margin and their counterparts in southeastern Greenland were unsuitably oriented to accommodate thinning of the crust during rifting of the northeastern Atlantic because the main structures strike parallel to the extension direction. In addition, their low-angle geometry was unsuitable to accommodate transform faulting. Thus, a significant delay should be expected for continental breakup at the Greenland–Iceland–Faroe Ridge compared with adjacent areas along the rift axis. Plume activity may very well be responsible for the intense magmatism recorded at the conjugate margin pair (southeastern Greenland and Faroe Islands) and in Iceland (Dahl-Jensen et al., 1997; Millett et al., 2015; Geoffroy et al., 2021; Jolley et al., 2021; Walker et al., 2022; Layfield et al., 2023). However, should the studied late Paleoproterozoic structures extend farther offshore than what is presented in Figure 2e, the anomalously thick character of the crust under the Greenland–Iceland–Faroe Ridge may then be explained by the existence of a (continuous?) rift-orthogonal ribbon (i.e., continuous orogenic bridge; Koehl and Foulger, 2024; Figure 6a–d) or by several isolated blocks of continental crust entrapped by extensive magmatism (e.g., rifted oceanic magmatic plateau; Coffin and Eldholm, 1992; Rime et al., 2024) or by several microcontinents similar to the Jan Mayen Microcontinent Complex and all rifted from one another and separated by regular, Penrose-like oceanic crust (e.g., Johnson and Heezen, 1967; Bott, 1985; Blischke et al., 2016). Since the thickness of the Greenland–Iceland–Faroe Ridge is consistently higher than 20 km from southeastern Greenland to the Faroe shelf, a scenario with several microcontinents separated by Penrose-like oceanic crust is unlikely. Ongoing work using seismic reflection data within the Greenland–Iceland–Faroe Ridge suggests that the late Paleoproterozoic structures described in the present study extend at least to eastern Iceland (Koehl et al., 2025) and, thus, that the Greenland–Iceland–Faroe Ridge may be a continuous orogenic bridge (Figure 6a–d; Koehl and Foulger, 2024), potentially developing into a rifted oceanic magmatic plateau (e.g., Rime et al., 2024), i.e., the early phase towards its evolution to a ruptured orogenic bridge. A continuous ribbon of continental crust between southeastern Greenland, Iceland, and the Faroe Islands is compatible with seafloor spreading at the Reykjanes Ridge and Kolbeinsey Ridge and with plume magmatism. The orogenic bridge may have formed during the late Paleoproterozoic Laxfordian–Nagssugtoqidian Orogeny (Figure 6a) and was then reworked and tightened during NW–SE-oriented Caledonian contraction (Figure 6b). The crust was then thinned during several ensuing episodes of extension, e.g., late–post-orogenic collapse in the Devonian and Permian–Triassic and Jurassic–Cretaceous rifting (Figure 6c). While relatively thinner crust northeast and southwest of Iceland was broken up, highly thickened orogenic crust at the Greenland–Iceland–Faroe Ridge is still being thinned and stretching together with seafloor spreading at the adjacent Reykjanes and Kolbeinsey ridges (Figure 6d; Koehl and Foulger, 2024).

 For **Figure 6** please refer ( https://s3-eu-west-1.amazonaws.com/openreseurope/linked/242354.18284_-_Figure_6_%28Comment_-_OREU%29.docx)

**Figure 6** Model showing the development of an orogenic bridge at the Greenland–Iceland–Faroe Ridge through (a–b) two orogeneses perpendicular to one another ((a) WNW–ESE-striking Laxofordian–Nagssugtoqidian Orogen and (b) NE–SW-striking Caledonian Orogen), (c) repeated extension along extensional faults and detachments and inherited orogenic structures, e.g., in the Devonian, Permian–Triassic, and Jurassic–Cretaceous, and (d) breakup along inherited, reactivated and/or overprinted orogenic structures in the Cenozoic. Note that any number of additional orogeneses and/or extensional collapse/rifting may be added in between these events. Abbreviations: GIFR: Greenland–Iceland–Faroe Ridge; TFZ: Tjörnes fault zone.”. Also updated the title of the “Implications for the Orogenic Bridge Theory” section into “Implications for the Orogenic Bridge Theory and breakup in the northeastern Atlantic”. Furthermore, added a new Figure 6 detailing the development of an orogenic bridge at the Greenland–Iceland–Faroe Ridge.  

**Comment 9:** One minor detail: in the plain language summary, on the fourth line from the bottom, ‘1.8 million years’ should read ‘1.8 billion years’.

**Response:** Agreed.

Changes: Replaced “million” by “billion” in the plain language summary.  

**Comment 10**: Actions: I leave it up to the authors to consider whether or not to act on my suggestions. However, I do feel that their assessment that the post-Caledonian tectonostratigraphic framework is 'irrelevant' to this study leaves the Discussion unbalanced and lacking crucial information that has an important bearing on the 'Timing of Formation...' and the ensuing history of reactivation. Nevertheless, I think that this is a very good paper with exciting consequences for better understanding the structural framework of the Faroe-Shetland continental margin.

**Response:** The authors of the present manuscript present their apologies for the misunderstanding created by their phrasing of the methods chapter. The authors of the present manuscript did not imply that post-Caledonian tectonostratigraphic framework is irrelevant to the study of the margin. This was also righteously pointed out by Prof. Krzywiec (reviewer 2) and was corrected accordingly. See also response to comments 1 to 7 and to Prof. Krzywiec’s comment 5.

Changes: See response to comments 1 to 7 and to Prof. Krzywiec’s comment 5 (reviewer 2).
